# Classifying Automated Driving Systems Using Telematics Data: A Comparative Study of GRU, LSTM, and Transformer Models

**DOI:** 10.3390/s26206570

**Published:** 2026-10-16

**Authors:** Bidhya Shrestha, Bhargab Acharya, Christos Papadopoulos

**Affiliations:** Department of Computer Science, University of Memphis, Memphis, TN 38152, USA; bacharya@memphis.edu (B.A.); cppdplos@memphis.edu (C.P.)

**Keywords:** automated driving systems, vehicle telematics, time-series classification, deep learning, GRU, LSTM, transformer encoder, robustness testing, intelligent transportation systems

## Abstract

The rapid advancement of sensing, artificial intelligence, and in-vehicle computing has accelerated deployment of automated driving systems (ADSs). As vehicles move toward software-defined architectures, multiple driving stacks—including manufacturer and aftermarket solutions—may coexist, raising the need to verify which system is actively controlling the vehicle from observable behavior alone. This paper investigates ADS identification using only harmonized vehicle telematics, without proprietary controller states or perception sensors. We compared three deep sequence models—Gated Recurrent Unit (GRU), Long Short-Term Memory (LSTM), and a Transformer Encoder—for distinguishing Comma Openpilot, Tesla Autopilot, Cadillac Super Cruise, and manual driving from eleven shared features over 32-timestep windows. On clean held-out data, all three architectures achieved strong performance (macro-F1 above 0.91; mean ± std over 20 random seeds), with the Transformer Encoder attaining the highest mean macro-F1 and the GRU providing competitive, lower-cost alternatives. We further evaluated robustness under a structured telematics noise framework covering six atomic threat families and four compound multi-fault conditions at different severity levels using a two-arm protocol over 7 random seeds: clean training (Arm A) versus evaluation matched noise-augmented training (Arm B). Under clean training, degradation was highly threat-dependent: *correlated cross-feature noise* and *stochastic drift*—and their compounds—caused the largest absolute and relative drops in macro-F1, whereas delay-with-hold *temporal jitter* and *temporal delay* had comparatively minor effects. This pattern is shared across GRU, LSTM, and Transformer Encoder models. Evaluation matched augmentation preserved clean-test accuracy while substantially reducing degradation, including at held-out severities and on compound faults withheld from training. The results show that telemetry-based ADS identification is feasible at scale and that reliable deployment requires explicit attention to continuous and cross-feature impairments.

## 1. Introduction

Automated driving systems (ADSs) are rapidly transforming modern transportation by enabling vehicles to perform increasingly complex driving tasks with limited human intervention. Recent advances in artificial intelligence, computer vision, sensor fusion, and vehicle control have accelerated the deployment of commercial ADS platforms like Tesla Autopilot [1], Cadillac Super Cruise [2], and aftermarket solutions such as Comma.ai Openpilot [3]. Although current commercial systems primarily operate at SAE Level 2 automation, the transition toward higher levels of autonomy is accelerating as we move toward software-defined vehicles (SDVs) [4]. In the near future, multiple software-based driving systems may coexist within the same transportation ecosystem and even the same vehicle specializing in different environments. Despite pursuing similar driving objectives, different ADS platforms exhibit unique behavioral characteristics due to differences in perception pipelines, control strategies, sensor configurations, and decision-making algorithms.

As vehicles become increasingly software-defined, reliably identifying the active driving system has become important for safety monitoring, cybersecurity, fleet management, insurance assessment, and regulatory compliance. Existing approaches typically rely on internal vehicle information, such as software status indicators, diagnostic messages, or manufacturer-provided system states. However, these sources may not always provide reliable evidence of actual vehicle behavior. Software failures, hardware malfunctions, cybersecurity attacks, or intentional modifications can cause discrepancies between reported system states and the true operational condition of the vehicle. “Dieselgate” [5] is a well-known example of such manipulation where a manufacturer altered ECU behavior when the vehicle was on the EPA test cycle to comply with regulatory checks. Therefore, independent, behavior-based verification mechanisms are needed to determine whether an ADS is active and which one, if multiple ADSs are present. Vehicle telematics provide a solid foundation for behavior-based ADS identification because they capture vehicle behavior without requiring access to proprietary software, perception sensors, or manufacturer-specific interfaces. Signals such as vehicle speed, acceleration, steering angle, steering rate, braking status, accelerator position, and turn indicators provide continuous information about the vehicle physical behavior. Since different ADS platforms implement distinct control policies, these temporal vehicle responses can serve as behavioral signatures for distinguishing among automated driving systems.

Recent advances in deep learning have significantly improved the analysis of sequential vehicle data. Recurrent architectures, including Long Short-Term Memory (LSTM) [6] and Gated Recurrent Unit (GRU) [7] networks, have been widely used for temporal modeling because of their ability to capture sequential dependencies in sensor measurements. More recently, Transformer-based architectures [8] have demonstrated strong performance in sequence modeling by employing self-attention mechanisms that capture relationships across entire input sequences while enabling efficient parallel processing. These architectures have been successfully applied to driver behavior recognition, trajectory prediction, anomaly detection, and vehicle state estimation. However, comparative evaluation of recurrent and Transformer architectures for identifying the underlying ADS platform solely from vehicle telematics—especially under structured telemetry degradation—remains limited.

Prior studies primarily focused on classifying human driving behavior rather than identifying automated driving systems. Saleh et al. [9] proposed a stacked LSTM model for classifying normal, aggressive, and drowsy driving behaviors using smartphone-based GPS, accelerometer, and gyroscope measurements. Their results demonstrated the capability of recurrent networks to model driving-related temporal patterns. However, smartphone-based sensing does not accurately nor completely capture the vehicle control behavior required for distinguishing among different ADS platforms. Enev et al. [10] used a combination of 15 sensors and a random forest classifier to identify 15 drivers and achieved an overall accuracy of 100%. However, the performance was not consistent across all driving environments. More specifically, the identification accuracy decreased to 91.33% in parking lot scenarios.

Vehicle-based telematics have also been explored for driver identification and behavior analysis. Girma et al.  [11] developed an LSTM-based driver identification framework using vehicle signals such as speed, engine RPM, throttle position, steering angle, and brake pedal displacement. Although their approach achieved strong classification performance and demonstrated robustness under noisy conditions, it relied on all available telemetry features without systematic feature selection. Furthermore, the robustness analysis introduced noise only to selected portions of the dataset, limiting the evaluation of realistic telemetry degradation.

Several studies have investigated the use of different sequence learning architectures for driving behavior recognition. Carvalho et al. [12] compared recurrent neural network variants, LSTM, and GRU models using smartphone accelerometer data for detecting driving events such as aggressive braking and lane changes. Their results showed that GRU achieved competitive performance while maintaining lower computational complexity. Similarly, Guo and Hansen  [13] demonstrated that Transformer Encoder can outperform recurrent architectures for driving behavior classification by leveraging self-attention to capture long-range temporal dependencies. However, these studies primarily considered limited-scale datasets and human driving behaviors, leaving open questions regarding their applicability to large-scale ADS identification.

Sensor faults in vehicles have been studied extensively in the fault diagnosis literature. Biddle and Fallah [14] proposed an SVM-based framework to detect, isolate, and identify drift, hard-over, erratic, spike, and stuck faults in the steering, braking, and torque sensors of an autonomous vehicle controller, while noting that noise-free simulated data likely inflates the reported accuracy. Abboush et al. [15,16] cataloged gain, offset, hard-over, stuck-at, delay, noise, packet loss, drift, and spike faults in automotive sensors and combined a denoising autoencoder with an LSTM to diagnose single and concurrent faults during hardware-in-the-loop testing. These studies focused on detecting and classifying the faults themselves; they do not examine how such faults affect downstream models that consume vehicle telemetry, such as behavior-based ADS identification.

Timing integrity has increasingly been recognized as a reliability concern in autonomous driving. Shahriar et al. [17] showed that multimodal perception pipelines that fuse camera and LiDAR are highly fragile to temporal misalignment introduced through the in-vehicle network, with even single-frame delays causing large drops in detection and tracking performance. Fan et al. [18] similarly addressed on-vehicle sensor staleness between LiDAR/radar and camera streams, and proposed staleness-aware fusion features together with realistic delay augmentation to maintain detection quality under asynchronous sensing. These studies established that cross-sensor latency and stale observations can break downstream autonomy stacks; however, they focused on perception fusion and control-relevant sensing rather than monitoring which automated driving system is active from telematics.

Robustness to realistic perturbations has recently received attention in both driving and general time-series modeling. Bench2Drive-Robust [19] evaluated closed-loop end-to-end driving policies under camera frame drops, occlusion, GPS and speed noise, and inference latency, finding that latency is the most damaging perturbation and that strong clean-condition performance does not imply robustness. Shift & Drift [20] perturbed the actuation commands of motion planners with uncorrelated Gaussian noise and temporally correlated Ornstein–Uhlenbeck noise, showing that correlated errors are more harmful because they were accumulated over time. Beyond driving, Wei et al. [21] benchmarked multivariate time-series anomaly detectors under Gaussian noise, channel dropout, and time shifts, and argued that absolute performance under perturbation is more informative than retention ratios. Hu and Ai [22] showed that randomly dropping input channels during training restored the robustness of forecasting models to noisy, misaligned, and missing co-variates. However, these studies evaluated the robustness of driving policies or generic time-series models; none addressed identifying which driving system is controlling a vehicle from telematics under realistic signal degradation.

Compared with driver behavior recognition, only a limited number of studies have investigated identification of the automated driving system itself. Maresca et al. [23] showed that autonomous and human-driven vehicles can be distinguished from camera clips and target-vehicle state in CARLA simulation, reaching approximately 93% accuracy, and reported modest degradation when state information was partially removed. Li et al. [24] extracted interpretable behavioral rules from roadside video using large language models to distinguish Waymo robotaxis from human-driven vehicles, achieving 90% accuracy. However, both studies address binary autonomous-versus-human classification of a single driving system from external observation, and neither evaluated robustness to realistic sensing degradation. Our previous work [25] demonstrated that GRU-based models can distinguish between multiple ADS platforms and manual driving using vehicle telematics from different driving systems. The study achieved weighted F1-scores above 90%, indicating that telematics contain distinctive behavioral patterns associated with different driving systems. However, the evaluation was limited to clean telemetry conditions and did not consider the impact of realistic sensor degradation, communication failures, or temporal synchronization errors that commonly occur in production vehicles.

Despite significant progress in sequential vehicle data modeling, four challenges remain. First, existing studies primarily focus on driver behavior recognition or driver identification rather than distinguishing among multiple ADS platforms. Second, evaluations are often performed on limited datasets, reducing confidence in their scalability to heterogeneous real-world driving environments. Third, existing ADS classification studies generally assume clean telemetry, while robustness against realistic sensor noise, calibration drift, and temporal jitter remains largely unexplored. Fourth, it is unclear whether training with synthetic corruption can recover robustness without sacrificing clean accuracy, or whether such gains transfer to unseen severities and compound faults.

To address these limitations, this paper presents a comprehensive evaluation of GRU, LSTM, and Transformer Encoder architectures for ADS identification using vehicle telematics alone under noisy conditions. We use the harmonized multi-platform dataset of [25], containing more than 3.4 million labeled telemetry samples collected from multiple ADS platforms, including Comma Openpilot, Tesla Autopilot, Cadillac Super Cruise, and manual driving. Along with evaluating classification performance under clean telemetry conditions, we introduce a structured robustness framework that systematically models realistic degradation affecting both continuous vehicle signals and binary event states. Through extensive sensitivity analysis, we quantify the impact of different noise mechanisms and severity levels on sequence learning architectures and identify the failure modes that most strongly affect ADS classification performance.

The main contributions of this paper are summarized as follows:

- We extend our earlier GRU-based ADS classifier [25] to a controlled comparison of GRU, LSTM, and causal Transformer Encoder models for identifying automated driving systems from vehicle telematics, without requiring access to proprietary ADS information.
- We introduce a structured telematics noise framework that models realistic degradation affecting both continuous sensor measurements and event-based vehicle signals.
- We perform an extensive sensitivity analysis across multiple noise types and severity levels to quantify the robustness of different sequence learning architectures.
- We show that noise-augmented training substantially reduces degradation under continuous and cross-channel noise, including at held-out severities and on compound faults withheld from training, without a significant loss of clean accuracy.

The remainder of this paper is organized as follows. Section 2 describes the datasets, feature selection, data preparation, noise framework, and model architectures. Section 3 presents the experimental results, including clean-data evaluation and the two-arm robustness analysis. Section 4 discusses the findings and limitations. Section 5 concludes the paper, and Section 6 summarizes the relationship to our previous publications.

## 2. Materials and Methods

This section describes the proposed framework for automated driving system classification using vehicle telematics and the methodology we used to evaluate model robustness under realistic telemetry degradation. The overall pipeline consists of six major stages: (1) ADS classification formulation, (2) dataset collection and harmonization, (3) feature selection and preprocessing, (4) deep sequence model development, (5) telemetry noise modeling and noise-augmented training, and (6) performance evaluation under clean and corrupted conditions.

### 2.1. Problem Formulation

We formulate ADS identification as a supervised multiclass sequence classification problem. Unlike conventional driver behavior recognition tasks that classify human driving styles, the objective of this study is to identify the active driving controller based solely on vehicle telematics.

Let $x_{t}\in R^{11}$ represent the telematics feature vector at timestep *t*, where each vector contains eleven features selected through the feature selection process. A temporal input sequence is represented as:

(1) $$X_{t}=\left[x_{t-T+1},\ldots ,x_{t}\right]\in R^{T\times 11}$$

where *T* denotes the sequence length. The classification objective is to learn a mapping:

(2) $$f:X_{t}\to y$$

where the output label is defined as:

y∈{0,1,2,3}

corresponding to Comma Openpilot, Manual Driving, Tesla Autopilot, and Cadillac Super Cruise, respectively. The goal is to identify the underlying driving system that generated the observed sequence of vehicle behaviors.

### 2.2. Data Sources

This study utilizes three real-world vehicle telematics datasets containing both automated driving and manual driving scenarios: the Comma2k19 dataset [26], the Cadillac Super Cruise dataset [27], and the Tesla Autopilot dataset [28]. These datasets were selected because they represent three distinct Level 2 automated driving systems developed by different manufacturers and software platforms.

The Comma2k19 dataset was collected using comma EON devices installed on two production vehicles, Toyota RAV4 and Honda Civic. The dataset contains more than 33 h of driving data collected on California Highway 280 between San Jose and San Francisco under diverse traffic and environmental conditions. It consists of approximately 12 million telemetry records with more than 140 vehicle-state features grouped into different events. The Cadillac dataset was collected from a 2019 Cadillac CT6 equipped with the Super Cruise automated driving system. The dataset includes approximately 60 highway driving sessions covering more than 1000 miles and contains 892,989 telemetry records. Similarly, the Tesla dataset was collected from a 2020 Tesla Model 3 equipped with the Autopilot system. It includes 60 driving sessions collected over approximately 1000 miles of suburban and highway driving, resulting in 1,320,598 telemetry records.

### 2.3. Feature Selection

Feature selection is an essential preprocessing step in machine learning because it identifies the most informative variables while removing irrelevant and redundant features. Selecting an appropriate feature subset improves model generalization, reduces computational complexity, and enhances interpretability.

The Cadillac, Tesla, and Comma datasets contain 129, 142, and 140+ features, respectively. For Comma dataset, we used only features from carstate event which describe various aspects of vehicle dynamics, including speed, acceleration, steering behavior, braking activity, and other control-related information. Since there were many features with redundant information, we utilized feature selection strategy to determine the minimum number of features that could classify the driving system.

We selected the Comma2k19 dataset as the reference dataset and applied TSFRESH (Time Series Feature Extraction based on Scalable Hypothesis Tests) [29] to identify relevant time-series features. TSFRESH performs automated feature selection using statistical hypothesis testing and controls the false discovery rate (FDR) through the Benjamini–Hochberg procedure. The TSFRESH analysis identified 22 relevant features, including *vEgo*, *rightBlinker*, *leftBlinker*, *brakeLights*, *standstill*, *steeringPressed*, *brakePressed*, *gasPressed*, *vEgoRaw*, *brake*, *gas*, *wheelSpeeds_rr*, *wheelSpeeds_rl*, *wheelSpeeds_fr*, *wheelSpeeds_fl*, *steeringTorque*, *doorOpen*, *aEgo*, *yawRate*, *seatbeltUnlatched*, *steeringRateDeg*, and *steeringAngleDeg*.

To verify the relevance of the selected features, we performed Mutual Information (MI) analysis  [30]. MI measures the statistical dependency between individual features and the target classification labels. The analysis confirmed that the TSFRESH-selected features contained the highest information regarding ADS classification. Subsequently, correlation analysis was performed to remove highly correlated features that provided redundant information. This process reduced the feature set from 22 to 17 features while preserving the most discriminative vehicle dynamics information.

The final 17-feature set consisted of *vEgo*, *rightBlinker*, *leftBlinker*, *brakeLights*, *standstill*, *steeringPressed*, *brakePressed*, *gasPressed*, *brake*, *gas*, *steeringTorque*, *doorOpen*, *aEgo*, *yawRate*, *steeringRateDeg*, *steeringAngleDeg*, and *seatbeltUnlatched*. From this reduced feature set, we identified 11 features that were available across all three datasets: *vEgo*, *aEgo*, *gas*, *standstill*, *brakePressed*, *yawRate*, *leftBlinker*, *rightBlinker*, *steeringAngleDeg*, *steeringRateDeg*, and *brakeLights*. These common features were used for the primary cross-dataset ADS classification experiments. Table 1 lists the 11 features common in all datasets, along with three additional feature that are common only to Comma2k19 and Cadillac datasets.

### 2.4. Data Preprocessing

Following feature selection, we performed additional preprocessing to ensure temporal and feature-level consistency among the three datasets. Since sequential deep learning models require uniformly sampled input sequences, all datasets were converted to a common sampling frequency of 10 Hz. TThe Tesla and Cadillac datasets already had a sampling frequency of 10 Hz, but the Comma2k19 dataset did not. Originally sampled at 100 Hz, the Comma2k19 dataset was downsampled by selecting the first sample from every ten consecutive observations. This approach reduced the sampling frequency while preserving the temporal characteristics required for sequence-based learning.

Feature representations were further standardized because identical physical measurements were recorded using different signal names and units across datasets. For example, vehicle speed was represented as *vEgo* in the Comma dataset, *VehicleSpeed* in the Cadillac dataset, and *Veh_speed* in the Tesla dataset. Although these signals represent the same physical quantity, they were recorded using different measurement units: *vEgo* in meters per second (m/s), *VehicleSpeed* in miles per hour (mph), and *Veh_speed* in kilometers per hour (kph). To ensure consistency, *VehicleSpeed* and *Veh_speed* were converted to m/s, and all vehicle speed measurements were renamed as *vEgo*. The same mapping procedure was applied to other common telemetry signals to maintain consistent feature names and units across all datasets. Table 1 summarizes the final features used in the experiments and their corresponding units.

### 2.5. Data Labeling

The classification labels were generated using vehicle-state indicators that specify whether the vehicle was operating under automated or manual control. Dataset-specific signals were used because each platform provides different indicators of automated driving status. Specifically, cruiseState.enabled was used for the Comma2k19 dataset, ACCCmdActive was used for the Cadillac dataset, and DAS_autopilotState was used for the Tesla dataset.

For the Comma2k19 and Cadillac datasets, an automated driving label was assigned when the corresponding indicator was True (1), whereas False (0) indicated manual driving. The Tesla dataset provides nine categorical states for DAS_autopilotState. We considered Autopilot to be active when the state was Active-Nominal, Active-Navigation, or Active-Restricted. All other states, including Disabled, Unavailable, Available, Fault, Aborting, and Aborted, were labeled as manual driving because the Autopilot system was not actively controlling the vehicle.

**Figure 1:**
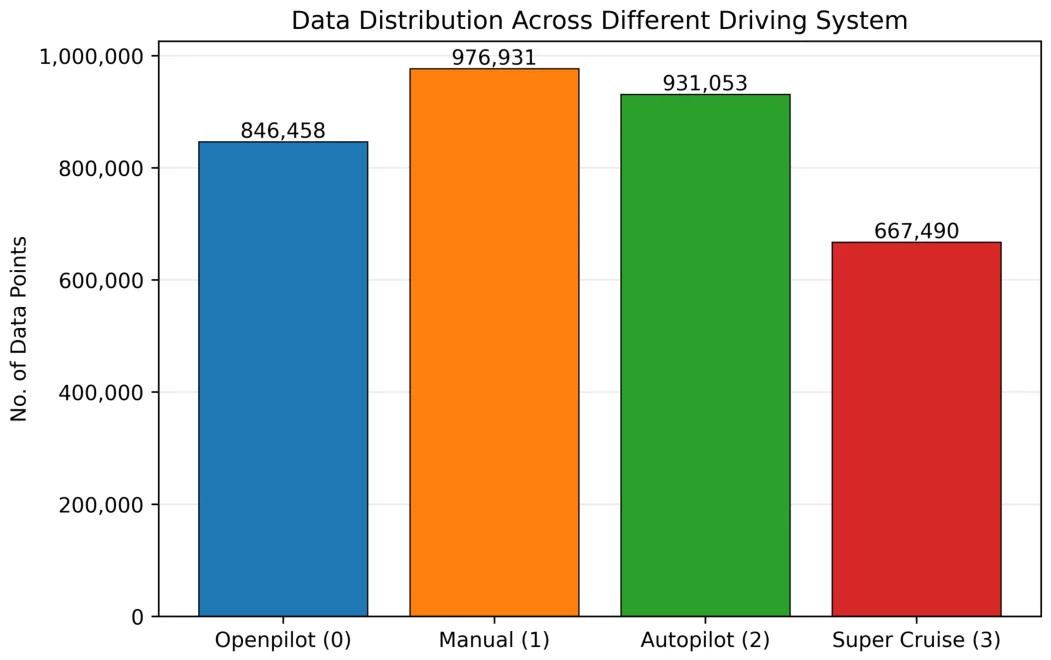
Sample distribution across four ADS classes.

After preprocessing and dataset integration, four class labels were assigned. Class 0 represents Comma Openpilot, Class 1 represents manual driving, Class 2 represents Tesla Autopilot, and Class 3 represents Cadillac Super Cruise. The final merged dataset contains 846,458 Openpilot samples, 976,931 manual driving samples, 931,053 Tesla Autopilot samples, and 667,490 Cadillac Super Cruise samples, as shown in Figure 1. This labeling strategy formulates ADS identification as a four-class sequence classification problem involving three automated driving systems and human-controlled driving.

### 2.6. Deep Sequence Learning Models

We evaluated three representative deep sequence learning architectures for ADS classification: Gated Recurrent Unit (GRU), Long Short-Term Memory (LSTM), and Transformer Encoder model. These architectures were selected because they represent different approaches for learning temporal dependencies from sequential vehicle telemetry. GRU and LSTM models capture temporal patterns through recurrent memory mechanisms, whereas the Transformer Encoder learns temporal relationships through self-attention.

All models received identical input representations consisting of sequences of 32 timesteps with 11 telematics features and performed four-class classification to identify the active driving mode: Comma Openpilot, manual driving, Tesla Autopilot, and Cadillac Super Cruise. The architectures were trained and evaluated using the same datasets, data splits, and optimization settings to enable a fair comparison.

#### 2.6.1. GRU Model

The Gated Recurrent Unit (GRU) is a recurrent neural network architecture that uses update and reset gates to regulate information flow through the hidden state. Compared with conventional recurrent neural networks, GRUs mitigate the vanishing gradient problem while maintaining a simpler structure and fewer parameters than LSTM networks. This enables efficient learning of temporal dependencies from vehicle telemetry sequences. The GRU classifier consists of six stacked GRU layers with a hidden dimension of 64. The hidden representation corresponding to the final timestep is passed through a fully connected layer to generate the four-class ADS prediction.

#### 2.6.2. LSTM Model

The Long Short-Term Memory (LSTM) network extends recurrent neural networks by introducing memory cells together with input, forget, and output gates. These gating mechanisms allow the model to selectively retain relevant information and discard unnecessary information, enabling effective modeling of long-term temporal dependencies. To ensure a consistent comparison with the GRU architecture, the LSTM model uses the same network depth and hidden dimension. The model consists of six stacked LSTM layers with a hidden size of 64, followed by a fully connected classification layer that produces the final ADS label.

#### 2.6.3. Transformer Encoder Model

The Transformer Encoder models sequential dependencies using self-attention instead of recurrent state updates. Unlike GRU and LSTM architectures, self-attention computes interactions among timesteps in parallel, which supports learning of both short-range and longer-range temporal structure within each window. In our implementation, each 11-dimensional telematics vector is linearly projected to a $d_{\mathrm{model}}=64$ embedding. Fixed sinusoidal positional encodings are then added to preserve temporal order. The resulting sequence is processed by six causal Transformer Encoder layers, each with eight attention heads and a feed-forward width of 256 ($4\times d_{\mathrm{model}}$). Causality is enforced with an upper-triangular attention mask so that timestep *t* attends only to positions ≤t, consistent with online telemetry classification. The representation at the final timestep is passed through a linear classifier to produce the four-class ADS prediction.

### 2.7. Telematics Noise Framework

Vehicle telematics collected in real-world environments are susceptible to various sources of degradation. For example, sensor measurement uncertainty, calibration errors, communication delays, packet transmission failures, and inconsistencies among related vehicle signals. Such imperfections can negatively affect the reliability of machine learning models used for ADS identification. Therefore, we developed a structured telematics noise framework to evaluate model robustness under realistic operating conditions. The noise strength was governed by an integer severity level L∈{1,2,3,4,5}, converted to a continuous scale factor s=L/5. Magnitude and probability parameters for each noise family followed an affine schedule in s. Table 2 reports the complete parameter schedule. Noise was applied to the standardized features, so all magnitudes ($\sigma_{\mathrm{noise}}$, $\sigma_{\mathrm{drift}}$, diag_var, and offdiag_cov) are expressed in units of one training-set standard deviation rather than in raw physical units.

The severity scale is a controlled synthetic ablation axis, in the discrete-tier tradition of common-corruption robustness benchmarks like in ImageNet-C [31], rather than a calibration to measured real-world fault statistics. Higher L produces a strictly larger perturbation within a threat family. L may not correspond to an equivalent real-world magnitude or incidence rate across different threat families or across architectures.

The proposed framework considers six noise families representing common failure modes in vehicle sensing and communication systems. These families are split by the physical nature of the channels they affect.

- Signal Noise: There are three types of signal noise that affects the continuous channel or features: *yawRate*, *steeringAngleDeg*, *steeringRateDeg*, *vEgo*, *aEgo*, *gas*. *Stochastic noise and drift* add independent Gaussian measurement noise $N(0,\sigma_{\mathrm{noise}}^{2})$ at every timestep, together with a cumulative random-walk bias formed by integrating independent increments $N(0,\sigma_{\mathrm{drift}}^{2})$ over the window, capturing sensor noise and slow calibration drift. Because the random walk accumulates, its standard deviation at the end of a 32-step window is $\sigma_{\mathrm{drift}}\sqrt{32}\approx 5.7\;\sigma_{\mathrm{drift}}$. *Temporal jitter* delays each continuous channel independently (per sample and channel) by Δ∼Uniform{0,…,J} frames with hold-at-edge filling, which repeats the earliest available value rather than wrapping, to model per-sensor reporting latency or relative inter-ECU clock skew. Consistent with sensor-staleness and temporal-misalignment formulations in multi-sensor autonomous driving [17,18], this one-sided delay-with-hold corruption treats timing faults as data age rather than circular reordering of the stream. *Correlated cross-channel noise* injects a multivariate Gaussian perturbation with equicorrelated covariance, with diagonal variance diag_var and off-diagonal covariance offdiag_cov, jointly within two physically coupled groups, (vEgo, aEgo, gas) and (yawRate, steeringAngleDeg, steeringRateDeg). This simulates a shared fault, such as electromagnetic interference or a biased state estimate, that affects related channels in a coordinated rather than independent manner.
- Event noise: There are three types of event noise that affects the binary channels or features: *leftBlinker*, *rightBlinker*, *brakePressed*, *brakeLights*, *standstill*. *Burst loss and spurious toggle* combine a two-state Markov freeze, which is entered with probability $p_{\mathrm{start}}$ and exited with probability $p_{\mathrm{end}}$, holding a channel at its previous value over contiguous frames, with independent per-frame bit flips at rate $p_{\mathrm{tog}}$. This models stuck or frozen switches together with transient bit errors. *Temporal delay* delays state transitions rather than the entire channel. When a binary flag changes, the new value is withheld for a random offset of up to $D_{\max}$ frames before being committed, while steady, non-transitioning intervals remain untouched. This represents ECU-to-ECU reporting lag concentrated at the moment a flag changes. *Cross-feature inconsistency* targets two logically paired signals, (leftBlinker, rightBlinker) and (brakePressed, brakeLights), and forces the second member of each pair to disagree with the first with probability $p_{\mathrm{inc}}$. This models desynchronization between ECUs that report related state. Severity L∈{1,…,5} scales all of the above through a shared affine schedule, with timing caps $J=D_{\max}=\mathrm{round}\left(L\right)$, so that the same graded corruption suite can be applied consistently across the GRU, LSTM, and Transformer Encoder models.
- Compound noise: To test generalization under simultaneous faults, the evaluation suite additionally applies four sequential two-family combinations at matched severity *L*: *stochastic+burst*, *jitter+delay*, *correlated+cross*, and *burst+delay*. The first three pair one continuous-channel (signal) family with one binary-channel (event) family acting on disjoint feature subsets, so the two corruptions do not interact; each compound is applied in a fixed order at the same *L*. *Burst+delay* instead composes two event-channel families on the same binary indicators: burst/freeze first, followed by *temporal delay* on the resulting channel. Hence it is order-sensitive by construction. This combination represents a single degrading discrete-signal source (e.g., a failing switch or desynchronized ECU) that produces both stuck values and delayed transition reporting.
  *Stochastic+burst* and *jitter+delay* are further motivated by identifiable shared physical mechanisms like electromagnetic interference affecting analog sensing and digital communication, and bus-timing congestion affecting continuous and discrete message delivery, respectively. Likewise, *correlated+cross* is included for combinatorial coverage across the remaining families rather than a specific shared-cause hypothesis.

Table 3 summarizes the noise models and their corresponding real-world manifestations.

### 2.8. Training Protocol and Robustness Arms

All deep sequence models were trained under a matched protocol so that differences in predictive performance can be attributed primarily to architecture. The merged dataset was partitioned into training, validation, and testing subsets using an approximately 70%–15%–15% chronological split performed independently within each data source to prevent temporal leakage. Complete segment_id groups were assigned to exactly one subset: the earliest segments by cumulative row count were used for training, the subsequent segments for validation, and the latest segments for testing. Sliding windows of fixed length T=32 were formed only within a segment and never crossed recording boundaries. Within each segment, windows were extracted with stride 1 (consecutive starts). The supervision label of each window was the ADS class at its final timestep; earlier timesteps provided temporal context only. This end-point labeling was used for training, validation, and testing. Feature standardization and class-weight estimation used the training subset exclusively. The training set was used for parameter optimization, the validation set guided early stopping, and the test set was reserved for final evaluation after checkpoint selection.

Models were trained with Adam, batch size 256, a maximum of 100 epochs, and early stopping on validation loss with patience 7; the best validation checkpoint was restored for testing. The loss was class-weighted cross-entropy, with weights computed from the training labels. Hyperparameters were selected by preliminary validation rather than exhaustive search: sequence lengths {4,8,16,32,64} and learning rates {0.1,0.01,0.001,0.0001} were compared, and T=32 with learning rate 0.0001 was fixed for all subsequent runs. All models in the main comparison were matched in depth and width (iso-depth and iso-width): each used six layers (stacked GRU layers, stacked LSTM layers, or Transformer Encoder layers) with a hidden or embedding dimension of 64 (H=64 for GRU/LSTM, d_model=64 for the Transformer). Clean-data experiments and the parameter-budget ablations (widened GRU/LSTM and narrower Transformer) used 20 random seeds with a fixed data partition. Because the full threat suite is computationally expensive, the robustness arms used 7 seeds with the same training settings and partition. Robustness was assessed under two training arms that differ only in corruption exposure during training. This design separates the impact of corruption on conventionally trained models from the extent to which corruption-aware augmentation mitigates that impact, while allowing the three architectures to be compared under identical training conditions.

Arm A (clean-trained) was trained exclusively on uncorrupted windows. This setup represents the conventional training scenario in which models are trained on curated, uncorrupted telemetry logs. It therefore provides the primary measure of noise impact, namely the degradation experienced when a clean-trained model is exposed to corrupted telemetry, without conflating architectural differences with the effects of augmentation.

Arm B (noise-augmented) used evaluation matched augmentation based on the six atomic evaluation families, while compound conditions were withheld from training. For each training batch, corruption was applied with probability 0.85; otherwise, the batch remained clean, providing approximately 15% clean exposure and limiting overfitting to noise-only inputs. When corruption was applied, exactly one threat family was sampled uniformly, and its severity *L* was drawn uniformly from {1,2,3}. Training was therefore deliberately restricted to the lower three levels of the full evaluation range, with L∈{4,5} excluded from training and all compound conditions withheld entirely. Consequently, higher-severity and multi-fault conditions evaluate out-of-distribution generalization rather than memorization of the most severe training conditions. Moreover, we also tested Arm B on the clean, uncorrupted test dataset to check whether evaluation matched augmentation imposed an accuracy cost on in-distribution data or remained approximately neutral. For each condition, we reported macro-F1 together with absolute and relative degradation computed against the same arm’s own clean-condition baseline for each seed.

### 2.9. Evaluation Metrics

We formulated ADS identification as a four-class classification problem, where each sample is assigned to one of four driving modes: Comma Openpilot, manual driving, Tesla Autopilot, or Cadillac Super Cruise. To evaluate classification performance comprehensively, we reported precision, recall, and F1-score for each individual class. Since the dataset exhibits class imbalance across the four classes, we used macro-averaged metrics as the primary evaluation criteria because they assign equal importance to each driving mode regardless of its number of samples.

Precision represents the proportion of correctly classified samples among all samples predicted as a specific class and is defined as:

(3) $$Precision=\frac{TP}{TP+FP}$$

where TP denotes the number of true positive predictions and FP denotes the number of false positive predictions.

Recall measures the ability of the classifier to correctly identify samples belonging to a specific class and is defined as:

(4) $$Recall=\frac{TP}{TP+FN}$$

where FN represents the number of false negative predictions.

The F1-score combines precision and recall using their harmonic mean:

(5) $$F1=2\times \frac{Precision\times Recall}{Precision+Recall}$$

For multiclass classification, the macro-F1 is calculated by averaging the F1-score values of all classes. This metric was selected as the primary evaluation measure because ADS identification requires reliable recognition of all driving modes, including classes with fewer samples. In addition to macro-F1, we reported class-specific precision, recall, and F1-score to provide detailed insight into model behavior.

We also recorded model complexity and deployment cost under a common measurement protocol. For each architecture we recorded (i) the number of trainable parameters, (ii) approximate floating-point operations (FLOPs) for a single forward pass over one input sequence of length T=32, computed as twice the number of multiply–accumulate operations, (iii) serialized model size (checkpoint footprint), (iv) wall-clock training time to the early-stopped checkpoint, (v) inference latency for batch size 1, and (vi) peak GPU memory consumption during training and inference. All timing and memory measurements were obtained on the same hardware and software stack (single NVIDIA GeForce RTX 4080 GPU (driver 610.60) using PyTorch 2.5.1 with CUDA 12.4.) to enable fair cross-model comparison.

To assess stability beyond a single training run, every deep model was evaluated over N=20 independent random seeds with a fixed data partition. For each metric m, we reported the sample mean and standard deviation over 20 seeds.

(6) $$\bar{m}=\frac{1}{N}\sum_{i=1}^{N}m_{i},\;s_{m}=\sqrt{\frac{1}{N-1}\sum_{i=1}^{N}{\left(m_{i}-\bar{m}\right)}^{2}},$$

Pairwise differences between the architectures were tested on seed-matched macro-F1 scores using the Wilcoxon signed-rank test with Holm correction for multiple comparisons. The overall differences among GRU, LSTM, and Transformer Encoder models were assessed with the Friedman test. Statistical significance was declared at α=0.05.

For robustness evaluation, we quantified performance degradation by measuring changes in macro-F1 under different noise types and severity levels. This enabled a direct comparison of the sensitivity of each deep learning architecture to realistic telematics impairments. The two-arm robustness evaluation (Arm A: clean training; Arm B: evaluation matched noise-augmented training), including atomic and compound corruptions at L=1,…,5 and the associated absolute/relative degradation metrics, was repeated over N=7 seeds under the same data-partition protocol, reflecting the higher computational cost of the full threat suite. All robustness means, $\Delta_{\mathrm{abs}}$, $\Delta_{\mathrm{rel}}$, and paired Arm A versus Arm B tests were therefore computed over these seven seeds.

### 2.10. Declaration on the Use of GenAI

During the preparation of this manuscript, GenAI was used solely for editorial assistance and improving clarity and readability. GenAI tools were not used for generating scientific content, designing experiments, collecting or analyzing data, interpreting results, or developing the methodology. The authors reviewed and verified all manuscript content and remain fully responsible for the accuracy and integrity of the published work.

## 3. Results

This section presents the experimental evaluation of the proposed ADS classification framework. We first establish baseline classification performance on clean telemetry (Section 3.1), and then evaluate the robustness of each architecture under structured telemetry noise (Section 3.2).

### 3.1. Clean Performance

All experiments in this subsection used clean training and test telematics data. We first compared the GRU, LSTM, and Transformer Encoder under the iso-depth/iso-width protocol, reporting overall metrics (Section 3.1.1) and class-wise behavior (Section 3.1.2). We then benchmarked the three models against conventional machine-learning baselines (Section 3.1.3) and compared their computational cost (Section 3.1.4) and training convergence (Section 3.1.5). Next, a parameter-budget ablation tested whether model capacity explains the observed differences (Section 3.1.6). Finally, we analyzed which telemetry features the models relied on (Section 3.1.7) and evaluated the effect of additional vehicle features introduced in our previous work [25] (Section 3.1.8).

#### 3.1.1. Performance Comparison on Clean Telemetry

We first evaluated the classification performance of the GRU, LSTM, and Transformer Encoder models using clean training and test telemetry data. This experiment established the baseline capability of each architecture to identify the active driving system under ideal telemetry conditions. We used identical training and evaluation protocols, a sequence length of 32 time steps, and 20 random seeds to compare the three iso-depth/iso-width sequence models on clean test data in terms of classification performance and computational cost.

Table 4 reports the clean-test classification performance of the three iso-depth/iso-width sequence models, with results presented as mean ± standard deviation over 20 random seeds. The Transformer Encoder achieved the highest mean values across all four metrics, with an accuracy of 0.934±0.007, macro-precision of 0.938±0.006, macro-recall of 0.942±0.006, and macro-F1 of 0.938±0.006. The GRU achieved an accuracy of 0.928±0.005, macro-precision of 0.932±0.005, macro-recall of 0.937±0.005, and macro-F1 of 0.932±0.005. The LSTM recorded the lowest mean values among the three models, with an accuracy of 0.913±0.007, macro-precision of 0.919±0.006, macro-recall of 0.923±0.007, and macro-F1 of 0.918±0.007.

We further evaluated whether the observed macro-F1 differences were statistically significant across the three architectures. A Friedman test on the per-seed macro-F1 scores indicated a statistically significant difference among the three models ($\chi^{2}=29.10$, $p=4.80\times 10^{-7}$). We therefore performed paired Wilcoxon signed-rank tests for all three model pairs and applied the Holm correction for multiple comparisons. The Transformer Encoder achieved a mean paired macro-F1 improvement of 0.0058 over the GRU (Holm-adjusted p=0.0153) and 0.0204 over the LSTM (Holm-adjusted $p=6.0\times 10^{-6}$). The GRU also achieved a higher macro-F1 than the LSTM, with a mean paired difference of 0.0146 (Holm-adjusted $p=8.0\times 10^{-6}$). All three pairwise differences remained statistically significant after multiple-comparison correction.

#### 3.1.2. Class-Wise Classification Performance

**Figure 2:**
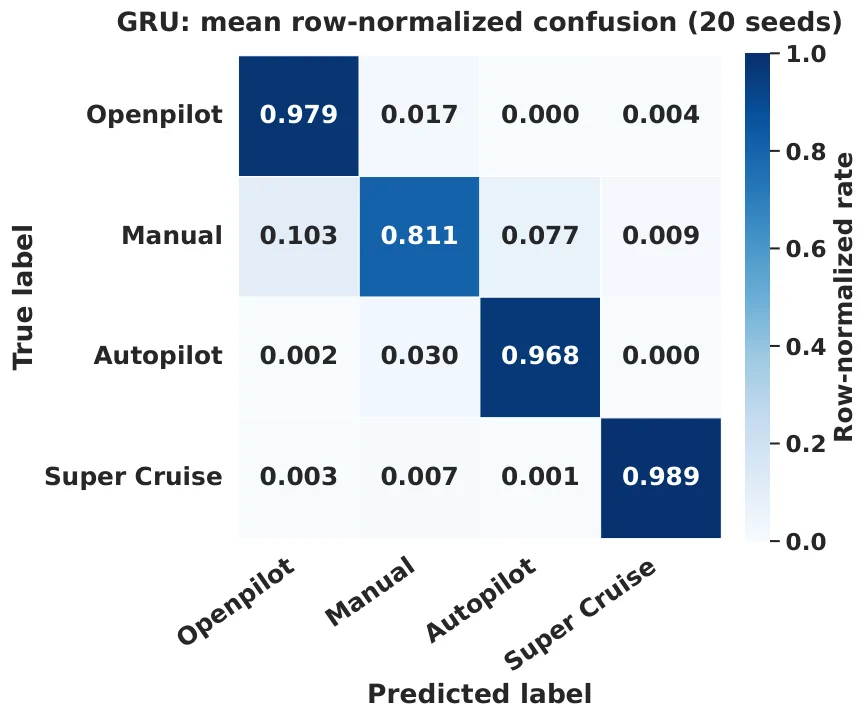
Mean row-normalized confusion matrix of the GRU (H=64) on the clean test set, averaged over 20 random seeds. Each cell (i,j) is the mean fraction of true class *i* predicted as class *j*; rows sum to one.

**Figure 3:**
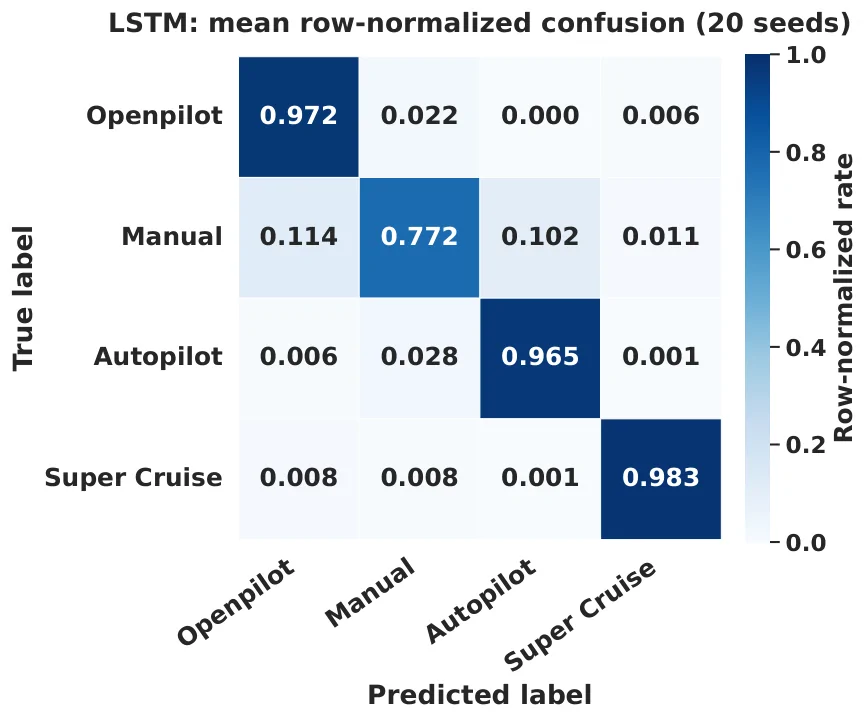
Mean row-normalized confusion matrix of the LSTM (H=64) on the clean test set, averaged over 20 random seeds. Each cell (i,j) is the mean fraction of true class *i* predicted as class *j*; rows sum to one.

**Figure 4:**
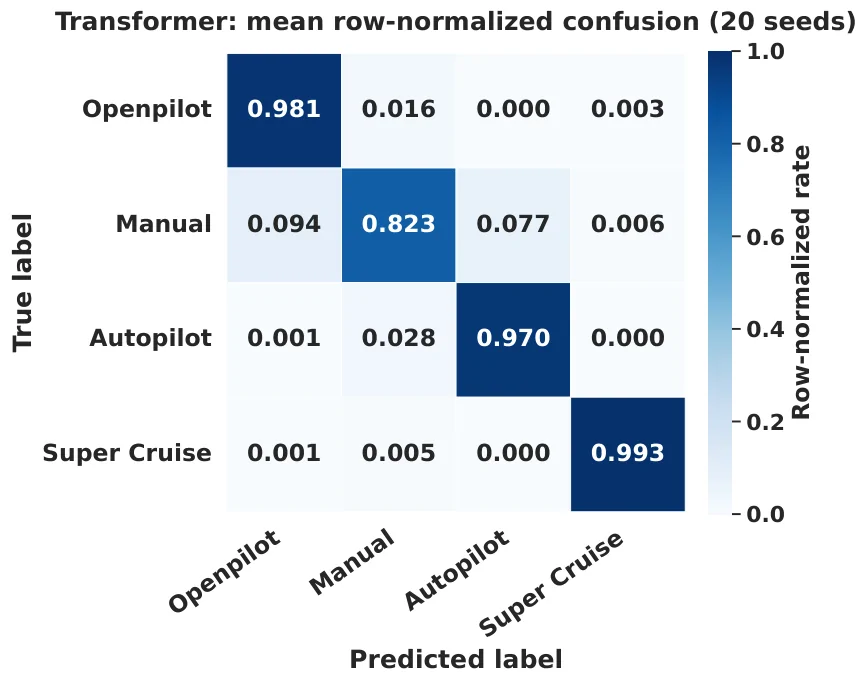
Mean row-normalized confusion matrix of the Transformer (d=64) on the clean test set, averaged over 20 random seeds. Each cell (i,j) is the mean fraction of true class *i* predicted as class *j*; rows sum to one.

Table 5 reports class-wise precision, recall, and F1-score for the four driving classes—Openpilot, Manual Driving, Autopilot, and Super Cruise—computed as means over the same 20 random seeds used in Table 4. Figure 2, Figure 3 and Figure 4 show the corresponding mean row-normalized confusion matrices. For each seed, a row-normalized confusion matrix is computed on the clean test set; each matrix entry is then averaged across the 20 seeds.

All three sequence learning architectures achieve strong class-wise performance, confirming that vehicle telematics contain sufficient temporal information to distinguish among different driving systems. The Transformer Encoder attains the highest overall macro-F1 (0.938), followed by the GRU (0.932) and the LSTM (0.918). This indicates that attention-based temporal modeling provides a modest but consistent advantage for clean-data ADS identification under the matched iso-depth/iso-width protocol.

Super Cruise is the most separable class for every architecture. It achieves the highest F1-scores (0.985 for GRU, 0.979 for LSTM, and 0.990 for Transformer), with mean recalls of 0.983–0.993. The corresponding diagonals in Figure 2, Figure 3 and Figure 4 are likewise the strongest entries in each matrix, and off-diagonal mass involving Super Cruise remains small. These results suggest that Super Cruise produces distinctive vehicle-control patterns that are readily separable from the other driving modes.

Autopilot and Openpilot are also recognized reliably. Mean F1-scores are 0.945/0.932/ 0.947 for Autopilot and 0.925/0.912/0.933 for Openpilot (GRU/LSTM/Transformer), with mean recalls above 0.96 for both ADS classes. Confusion among the automated classes themselves remains limited: the off-diagonal ADS-to-ADS rates in Figure 2, Figure 3 and Figure 4 are typically only a few percent or less, and are smallest for the Transformer.

Manual Driving remains the most challenging class for all models. Although precision is high (0.946 for GRU, 0.941 for LSTM, and 0.950 for Transformer), mean recall is substantially lower (0.811, 0.772, and 0.823, respectively). Thus, when the models predict Manual they are usually correct, but a non-trivial fraction of true Manual windows is assigned to an automated driving label. Figure 2, Figure 3 and Figure 4 show that these errors concentrate in the Manual row and are directed primarily toward Openpilot and Autopilot rather than Super Cruise. On average, 10.3%, 11.4%, and 9.4% of Manual windows are predicted as Openpilot, and 7.7%, 10.2%, and 7.7% as Autopilot, for GRU, LSTM, and Transformer, respectively. This pattern is consistent with human driving occasionally resembling Level-2 longitudinal and lateral control in terms of vehicle dynamics. Relative to the recurrent models, the Transformer achieves the highest Manual recall (0.823) and the lowest Manual→Openpilot rate (0.094), matching its higher Manual F1 in Table 5.

Overall, the seed-averaged class-wise metrics and confusion matrices support three conclusions. First, clean telematics enable reliable identification of all four driving modes. Second, Super Cruise is easiest to separate. Third, Manual Driving is the dominant residual error source through confusion with Openpilot and Autopilot.

#### 3.1.3. Comparison with Conventional Machine-Learning Baselines

To provide a reference point for conventional machine-learning approaches, we evaluated Random Forest [32] and XGBoost [33] using the same 32-step window protocol used for the sequence models. For these baselines, each 32-step window was flattened into a 352-dimensional feature vector before classification. Random Forest used 200 trees, unlimited depth, min_samples_leaf =1, and class_weight = balanced. XGBoost used up to 500 trees (max_depth =6, learning rate 0.1, subsample 0.8, colsample_bytree =0.8, λ=1.0), the hist tree method, and early stopping with patience 30 on validation multiclass log-loss. Both models were evaluated over the same 20 random seeds and fixed data partition, and Table 6 reports the mean ± standard deviation of clean-test accuracy, macro-precision, macro-recall, and macro-F1.

Among the two conventional baselines, XGBoost outperforms Random Forest across all four metrics. XGBoost achieves an accuracy of 0.9136±0.0003 and a macro-F1 of 0.9179±0.0003, compared with 0.9035±0.0002 accuracy and 0.9078±0.0002 macro-F1 for Random Forest. The same ordering holds for macro-precision and macro-recall (0.9196±0.0003 and 0.9246±0.0003 for XGBoost versus 0.9090±0.0001 and 0.9139±0.0001 for Random Forest). The extremely small seed-to-seed standard deviations for the tree models reflect near-deterministic training under a fixed split; they are therefore not comparable to the deep models’ seed variance, which includes random weight initialization and optimizer stochasticity. We report these ± values for completeness, but interpret deep versus tree comparisons primarily through mean scores under the shared protocol rather than through variance magnitude. Relative to the sequence models, XGBoost attains a mean macro-F1 comparable to the LSTM (0.9179 versus 0.918). The GRU and Transformer remain higher (0.932 and 0.938), corresponding to gaps of approximately 1.4 and 2.0 percentage points relative to XGBoost. A similar ordering appears for macro-precision and macro-recall: GRU 0.932/0.937 and Transformer 0.938/0.942, versus 0.9196/0.9246 for XGBoost. These results provide a comparison between sequence-based models and conventional classifiers operating on flattened telemetry windows. XGBoost is a strong flattened-window baseline and matches LSTM-level mean performance, indicating that substantial predictive information remains even without explicit sequential modeling. The higher mean macro-F1 of the GRU and Transformer suggests that sequence models extract additional predictive structure under this protocol.

#### 3.1.4. Computational and Resource Comparison

We next compared the model complexity and computational requirements of the GRU, LSTM and Transformer Encoder to characterize the trade-off between classification performance and resource usage. The Transformer Encoder (d=64, n_layers=6) achieves the highest mean macro-F1 of 0.938±0.006. It has 300,932 trainable parameters, a 1.24 MB checkpoint, a mean training time of 1098 s, a batch-1 inference latency of 1.44 ms, peak training GPU memory of approximately 270 MB, and peak batch-1 inference GPU memory of 34 MB. The GRU (H=64, n_layers=6) is the most compact and fastest at inference, with 139,844 parameters, a 0.56 MB checkpoint, a mean training time of 932 s, a batch-1 latency of 0.74 ms, peak training GPU memory of 163 MB, and peak batch-1 inference GPU memory of 25 MB, while remaining competitive in accuracy (macro-F1 0.932±0.005). The LSTM (H=64, n_layers=6) has 186,372 parameters, a 0.75 MB checkpoint, the shortest mean training time (812 s), a batch-1 latency of 0.90 ms, peak training GPU memory of 158 MB, and peak batch-1 inference GPU memory of 28 MB. Its macro-F1 of 0.918±0.007 is the lowest among the three models.

These results show a clear accuracy–efficiency trade-off under the matched iso-depth/iso-width protocol. Relative to the GRU, the Transformer uses approximately 2.2× more parameters, about 2.3× more FLOPs per sequence, about 1.7× more peak training GPU memory, nearly 2× the batch-1 latency, and moderately higher batch-1 inference memory (34 MB vs. 25 MB). The GRU therefore matches Transformer macro-F1 within about 0.6 points at substantially lower parameter count, latency, and training memory. The LSTM uses the least peak training GPU memory and the shortest mean training time, but its higher latency and lower macro-F1 make it a weaker accuracy–efficiency compromise than the GRU.

Table 7 summarizes the model complexity, computational cost, and clean-test classification performance of the three architectures. Overall, the comparison shows that the Transformer Encoder provides the highest classification performance at the cost of greater resource requirements, whereas the GRU provides a lower-resource alternative with a relatively small reduction in macro-F1. The LSTM has the lowest mean training time and slightly lower peak GPU memory usage than the GRU, but its lower classification performance results in a larger performance gap relative to the other two architectures.

#### 3.1.5. Training Convergence Analysis

**Figure 5:**
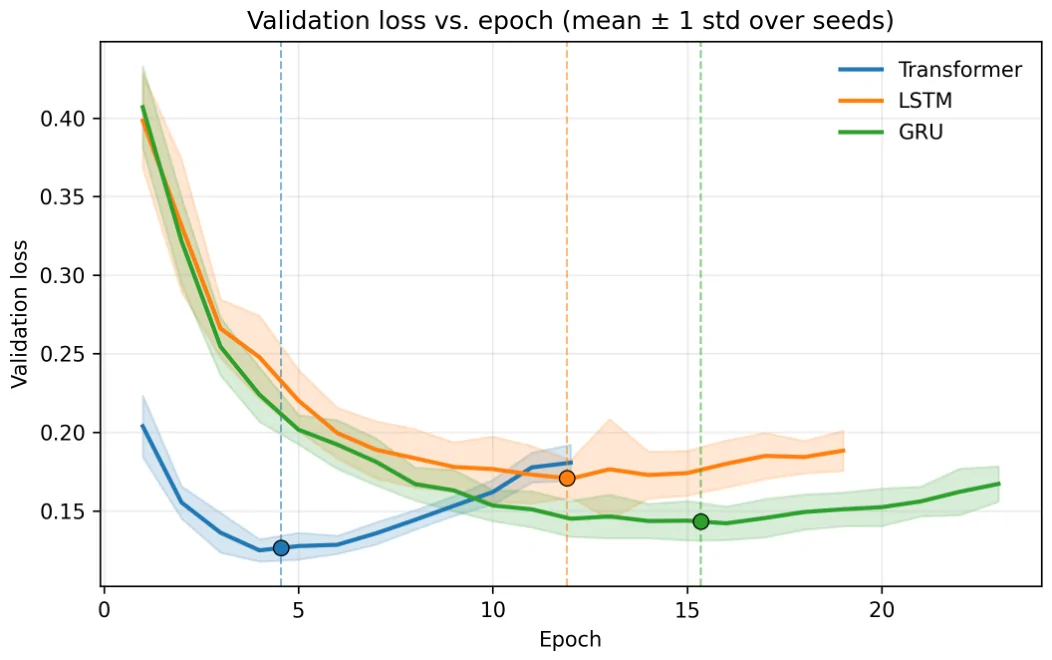
Training convergence of the iso-depth/iso-width models over 20 random seeds. Mean validation loss versus epoch for the GRU, LSTM, and Transformer Encoder; shaded bands show ±1 standard deviation across seeds. Because of early stopping (patience of 7 epochs), curves are shown only for epochs with at least 10 active seeds. Vertical dashed lines mark the mean best validation-loss epoch across seeds (Transformer 4.6, LSTM 11.9, GRU 15.4).

Figure 5 compares training convergence of the GRU, LSTM, and Transformer Encoder under the matched training protocol. Early stopping (patience of 7 epochs) selects the lowest-validation-loss checkpoint for each seed, and test metrics are reported only for that checkpoint (Table 4).

The Transformer Encoder reaches its best checkpoint in the fewest epochs: per-seed best epochs average 4.6±0.9, compared with 11.9±2.1 for the LSTM and 15.4±2.3 for the GRU. Its mean validation loss falls sharply during the first epochs and remains the lowest of the three models through the early shared-epoch regime (approximately epochs 1–8). It then rises slightly, indicating that the model begins to overfit after the selected checkpoint. Because Transformer epochs are about twice as long, the gap in time to the best checkpoint is smaller (≈440 s, 510 s, and 640 s for the Transformer, LSTM, and GRU), although the Transformer still reaches it first. Earlier convergence does not imply better final performance. The LSTM reaches its best checkpoint earlier than the GRU but attains lower macro-F1, whereas the GRU continues to improve for more epochs and reaches competitive performance. Per-seed best validation loss is 0.121±0.005 for the Transformer, 0.136±0.007 for the GRU, and 0.163±0.011 for the LSTM.

#### 3.1.6. Parameter-Budget Ablation Study

The iso-depth/iso-width comparison uses the same number of layers and hidden dimensions across the recurrent models and the Transformer, but it does not equalize their parameter counts. The Transformer Encoder contains approximately 301 k parameters, compared with 140 k for the GRU and 186 k for the LSTM. To determine whether the observed performance differences are primarily attributable to this difference in model capacity, we conducted three additional parameter-budget ablation experiments using the same 20-seed training and evaluation protocol. Specifically, we increased the GRU hidden dimension from H=64 to H=94, increased the LSTM hidden dimension from H=64 to H=82, and reduced the Transformer embedding dimension from d=64 to d=48.

Table 8 summarizes parameter counts, computational measurements, and clean-test performance. We treat the number of parameters, FLOPs per input sequence, peak training GPU memory, and batch-1 inference latency as the primary computational-cost measures. Wall-clock training time is reported for completeness but depends on both mean time per epoch and the number of epochs completed under early stopping; therefore, lower Train (s) does not by itself imply lower compute intensity.

Increasing the GRU hidden dimension from H=64 to H=94 increased the parameter count from 139,844 to approximately 298 k, closely matching the 300,932 parameters of the Transformer Encoder. However, the larger GRU did not improve classification performance. Its macro-F1 decreased slightly from 0.932±0.005 to 0.930±0.007, while accuracy decreased from 0.928±0.005 to 0.926±0.007. The paired macro-F1 difference was approximately −0.003, and the Wilcoxon signed-rank test did not indicate a statistically significant difference (p=0.15). On the cost side, FLOPs rose from 8.79M to 18.86M and peak training GPU memory from 163 MB to 247 MB, but batch-1 inference latency decreased slightly (0.74 ms to 0.68 ms). Mean wall-clock training time also decreased (932 s to 697 s), mainly because early stopping triggered after fewer epochs on average (22.4 vs. 16.5); mean time per epoch remained nearly unchanged (41.7 s vs. 42.4 s).

We next reduced the Transformer embedding dimension from d=64 to d=48, reducing the parameter count from 300,932 to approximately 170 k. Although the reduced model remained larger than the GRU H=64 configuration, its parameter count was substantially closer to that of the GRU. The Transformer d=48 achieved a macro-F1 of 0.936±0.007, compared with 0.938±0.006 for the original d=64 configuration. The paired macro-F1 difference was approximately −0.002, with no statistically significant difference (p=0.47). Relative to GRU H=64, the reduced Transformer still required more resources on several axes (11.83M FLOPs, 281 MB peak training GPU memory, and 1.35 ms batch-1 latency vs. 8.79M, 163 MB, and 0.74 ms). Relative to Transformer d=64, parameters, FLOPs, and batch-1 latency decreased, but measured training cost did not: mean wall-clock training time increased from 1098 s to 1397 s, reflecting both a higher mean time per epoch (95.0 s vs. 106.3 s) and more epochs before early stopping (11.6 vs. 13.2). Peak training GPU memory was also slightly higher (281 MB vs. 270 MB). Thus, fewer parameters do not imply uniformly lower training cost under this protocol.

Finally, we increased the LSTM hidden dimension from H=64 to H=82, increasing its parameter count from 186,372 to approximately 304 k and thereby closely matching the Transformer parameter budget. The larger LSTM achieved a macro-F1 of 0.915±0.009, compared with 0.918±0.007 for the original LSTM. The paired macro-F1 difference was approximately −0.002, and the Wilcoxon test indicated no statistically significant difference (p=0.50). Despite having approximately the same number of parameters as the Transformer, the LSTM H=82 achieved a substantially lower macro-F1 compared with 0.938±0.006 for the Transformer. Cost changes mirrored the GRU ablation: FLOPs and peak training GPU memory increased (11.72 M to 19.17 M; 158 MB to 217 MB), while batch-1 latency decreased (0.90 ms to 0.68 ms). Mean training time fell from 812 s to 733 s mainly because fewer epochs were completed (18.9 vs. 16.9), with mean time per epoch nearly unchanged (43.0 s vs. 43.3 s).

Overall, the parameter-budget ablations show that increasing the capacity of the GRU or LSTM does not improve clean-test macro-F1, while reducing the Transformer parameter budget from approximately 301 k to 170 k parameters does not produce a statistically significant change in performance. These results indicate that parameter count alone does not explain the performance differences observed in the iso-depth/iso-width comparison. They also show that computational cost is not monotone in parameter count: widening the recurrent models increases FLOPs and peak training memory but can reduce measured latency and early-stopped wall-clock training time, whereas narrowing the Transformer reduces parameters and FLOPs but can increase training time. Within the parameter ranges evaluated in this study, the results therefore suggest that architectural differences contribute to the observed performance differences beyond model size alone.

#### 3.1.7. Feature Importance Analysis

To interpret which telemetry signals most influence ADS-mode classification, we estimated global feature importance using permutation importance [32] on the held-out clean test set for the trained GRU, LSTM, and Transformer Encoder models under the same leakage-aware, segment-complete evaluation protocol used for the main results. For each input feature, we randomly shuffled that feature across test-split rows while keeping all other inputs unchanged, reconstructed only within-segment windows, and measured the resulting decrease in macro-F1 relative to the unperturbed baseline. A larger F1 drop indicates greater model reliance on that feature. Importance estimates were averaged over five independent permutations.

Table 9 ranks features by their mean macro-F1 drop across the three architectures. Predictions are dominated by four physically meaningful control and dynamics signals: *gas*, *steeringAngleDeg*, *brakePressed*, and *yawRate*. These encode longitudinal actuation and lateral motion, which are closely tied to ADS operating states. Event indicators (*blinkers*, *standstill*) and *steeringRateDeg* generally produce smaller drops, suggesting limited unique contribution once the primary continuous and brake/throttle cues are available. The low *steeringRateDeg* importance is consistent with redundancy with steering angle and yaw rate. This holds most clearly for the GRU and Transformer; for the LSTM, standstill and the blinkers (0.19–0.23) still exceed *aEgo* (0.17) and *steeringRateDeg* (0.10).

Per-model rankings differ. The GRU relies most on *gas* (0.66), followed by *steeringAngleDeg* and *brakePressed* (≈0.38 each). The Transformer also ranks *gas* first (0.61), followed by *steeringAngleDeg* (0.48), *vEgo* (0.44), and *yawRate* (0.43); *brakePressed* ranks fifth (0.34). The LSTM assigns its largest drop to *brakePressed* (0.64), followed by *gas*, *yawRate*, and *steeringAngleDeg*. Thus, three of the four top features are common to all models, with the Transformer substituting *vEgo* for *brakePressed*. All three models rely mainly on core control and dynamics signals, supporting the physical plausibility of the learned representations.

#### 3.1.8. Effect of Additional Vehicle Features

The primary experiments used the 11 telemetry features common to all three datasets to enable a fair comparison across the Comma Openpilot, Tesla Autopilot, and Cadillac Super Cruise platforms. However, the Comma2k19 and Cadillac datasets share three additional telemetry features that are unavailable in the Tesla dataset, namely steeringPressed, steeringTorque and brake. To investigate whether these additional signals provide further discriminative information, we performed a supplementary experiment using the GRU model with a 14-feature input representation.

The inclusion of the three additional telemetry features improved the macro-F1 to 0.97 for all three classes as shown in Table 10. This indicates that richer vehicle-state information provides additional cues for distinguishing driving modes. Nevertheless, these features are unavailable in the Tesla dataset and therefore cannot be incorporated into the unified three-dataset evaluation. Consequently, the primary experiments throughout this paper were conducted using the common 11-feature representation to ensure a fair and consistent comparison across all ADS platforms.

### 3.2. Robustness Evaluation of GRU, LSTM, and Transformer Encoder Models

To evaluate model reliability under realistic operating conditions, we assessed the three architectures under the telemetry noise framework of Section 2.7 across the two training regimes of Section 2.8. We first characterized the vulnerability of clean-trained models (Arm A, Section 3.2.1), then evaluated the mitigation provided by noise-augmented training (Arm B, Section 3.2.2), and finally verified that this augmentation did not compromise clean-test accuracy (Section 3.2.3).

#### 3.2.1. Robustness Evaluation Under Clean Training (Arm A)

This subsection characterizes the vulnerability of sequence classifiers trained exclusively on uncorrupted telemetry. Arm A models (GRU, LSTM, and Transformer Encoder) were optimized on clean sliding windows of length T=32 and then evaluated on a fixed stress suite comprising the clean test split, six atomic threat families at severities L=1,…,5, and four compound (multi-fault) conditions at the same severity levels. Because no synthetic noise was introduced during training, the observed degradations quantify the cost of deploying a nominally accurate clean-trained model under realistic telemetry impairments. All scores are means over seven random seeds which is distinct from the 20-seed clean evaluation in Table 4.

To provide a unified robustness view, we reported three complementary quantities for each condition: (i) absolute macro-F1; (ii) the absolute drop from that arm’s own clean baseline,

(7) $$\Delta_{\mathrm{abs}}=F1_{\mathrm{clean}}-F1_{\mathrm{corrupt}},$$

and (iii) the relative degradation percentage,

(8) $$\Delta_{\mathrm{rel}}=100\times \frac{\Delta_{\mathrm{abs}}}{F1_{\mathrm{clean}}}.$$

**Figure 6:**
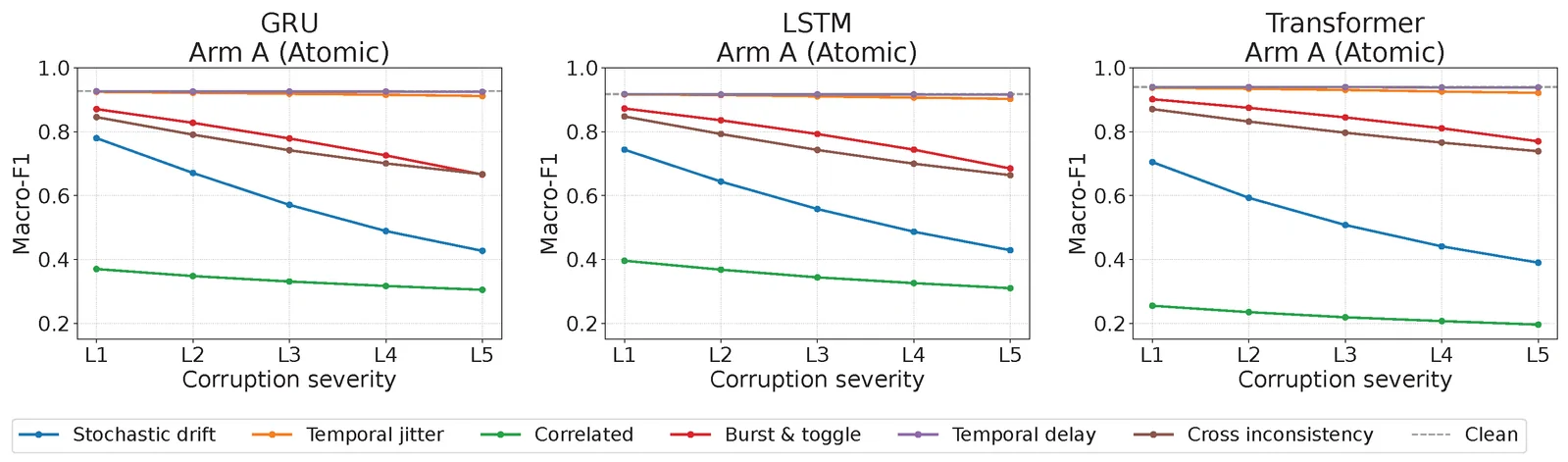
Macro-F1 versus corruption severity for clean-trained models (Arm A) under atomic threats. Panels (left–right): GRU, LSTM, and Transformer Encoder. The dashed line denotes each model’s clean-test macro-F1.

**Figure 7:**
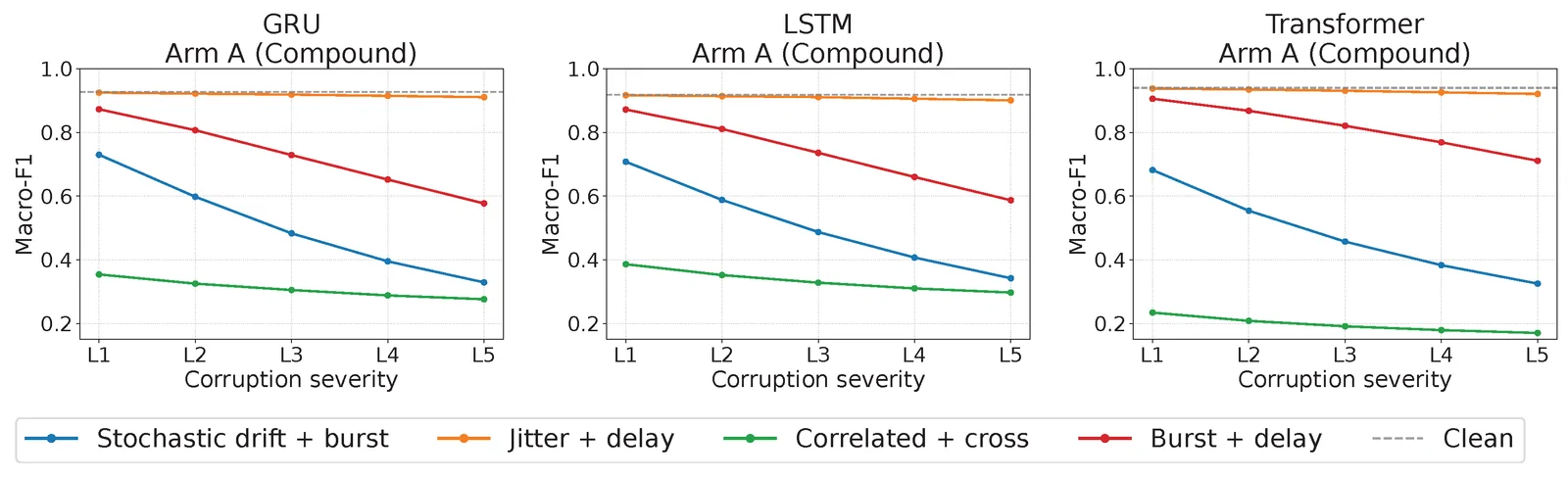
Macro-F1 versus corruption severity for clean-trained models (Arm A) under compound (multi-fault) threats. Panels (left–right): GRU, LSTM, and Transformer Encoder. The dashed line denotes each model’s clean-test macro-F1.

Both drops are computed per seed against Arm A’s clean-condition score for that seed and then averaged. Figure 6 plots mean macro-F1 versus severity for the six atomic families (one panel per architecture). Figure 7 shows the corresponding curves for the four compound conditions. Together, the figures make the severity trends visible, while Table 11 reports the unified F1/$\Delta_{\mathrm{abs}}$/$\Delta_{\mathrm{rel}}$ triple at L=5.

On clean test data, Arm A performance is strong and comparatively homogeneous: Transformer Encoder mean macro-F1 is 0.940, GRU 0.927, and LSTM 0.918. By definition, $\Delta_{\mathrm{abs}}=0$ and $\Delta_{\mathrm{rel}}=0$ on this clean condition because the clean score is the baseline used for all subsequent stress metrics. These baselines establish that all three models learn effective in-distribution decision rules; the stress curves below show that clean accuracy alone is a poor proxy for robustness.

Across all three panels, the curves separate into two regimes. *Temporal jitter* and *temporal delay* remain nearly flat from L=1 to L=5, staying close to each model’s clean baseline. At L=5, relative degradation remains below 2.1% for *Temporal jitter* and at most 0.2% for *temporal delay*, so absolute macro-F1 stays high (e.g., GRU 0.912/0.925; Transformer 0.922/0.939). In contrast, continuous-signal threats produce steeply declining trajectories: *stochastic drift* fall sharply as severity increases, with the largest vertical gaps appearing between L=1 and L=3 and continued decline through L=5. On the other hand, *correlated noise* drops sharply already at L=1 and only declines modestly thereafter. *Stochastic drift* yields $\Delta_{\mathrm{rel}}\approx 53$–58% (F1 0.390–0.429), while *correlated noise* reaches $\Delta_{\mathrm{rel}}=67.0\%$ (GRU), 66.2% (LSTM), and 79.1% (Transformer), with absolute drops of 0.621, 0.608, and 0.744, respectively. *Burst & toggle* and *cross-inconsistency* form an intermediate band: their curves slope downward more gently than *correlated* or *stochastic drift* but remain clearly below the flat *Temporal jitter/Temporal delay* lines ($\Delta_{\mathrm{rel}}\approx 18$–28% at L=5).

The three architectures showed the same qualitative pattern: flat timing curves and steeply declining continuous-noise curves. The Transformer Encoder’s *correlated-noise* curve was the lowest of the three at every severity (0.255 at L=1), even though its clean macro-F1 was the highest. The harmful families declined monotonically with L, confirming that the severity schedule imposes graded stress.

Compound panels reinforce the atomic ranking when two faults co-occur on the same window. Curves for *correlated+cross* and *stochastic drift+burst* track the severe continuous parents and sit at the bottom of each plot: at L=5, $\Delta_{\mathrm{rel}}$ reaches 70.2%/67.6%/81.9% for *correlated+cross* and approximately 63–65% for *stochastic+burst*. *Burst+delay* declines to an intermediate level (GRU/LSTM $\Delta_{\mathrm{rel}}\approx 36$–38%; Transformer 24.4%). *Jitter+delay* remains nearly flat and close to the clean baseline ($\Delta_{\mathrm{rel}}\approx 1.7$–2.1%), consistent with the weak individual effects of *temporal jitter* and *temporal delay* in Figure 6.

At L=5, relative degradation is largest for *correlated+cross* and *correlated noise*, followed by *stochastic drift+burst* and *stochastic drift*. *Temporal jitter*, *temporal delay*, and *jitter+delay* remain the smallest. *Stochastic drift* and *stochastic drift+burst* decline progressively with severity, whereas *correlated noise* and *correlated+cross* drop sharply at L=1 and change only modestly thereafter. Timing threats stay nearly flat across L=1,…,5. The same threat ordering appears for GRU, LSTM, and Transformer. These large losses under clean training motivate the comparison with noise-augmented training in Section 3.2.2.

#### 3.2.2. Robustness Evaluation Under Noise-Augmented Training (Arm B)

**Figure 8:**
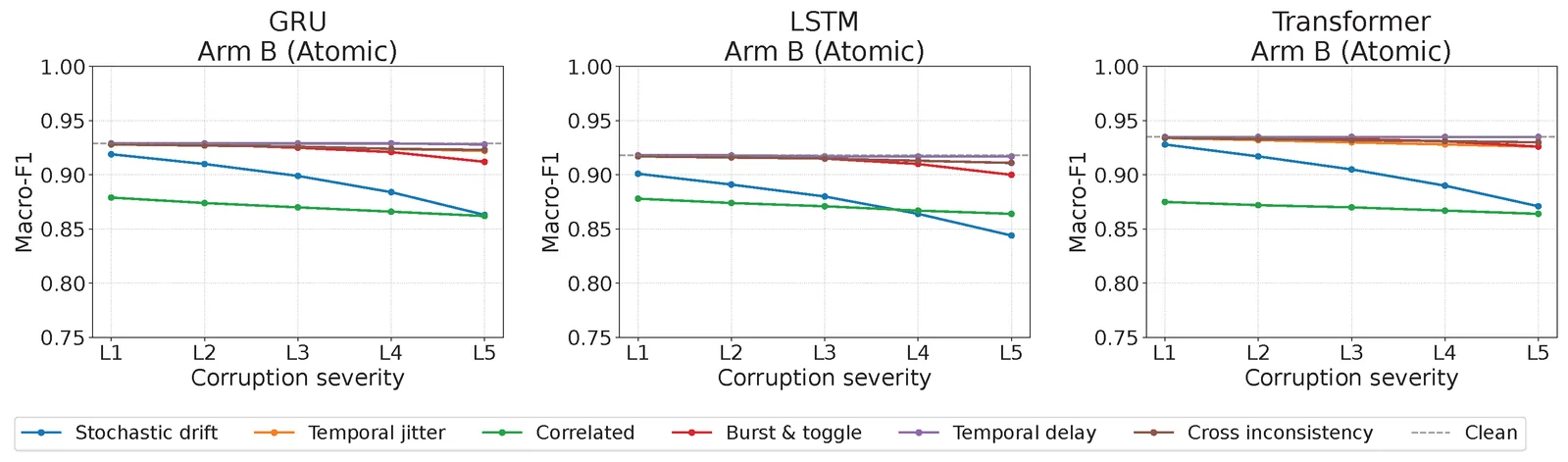
Macro-F1 versus corruption severity for noise-augmented models (Arm B) under atomic threats. Panels (left–right): GRU, LSTM, and Transformer Encoder. The dashed line denotes each model’s clean-test macro-F1.

**Figure 9:**
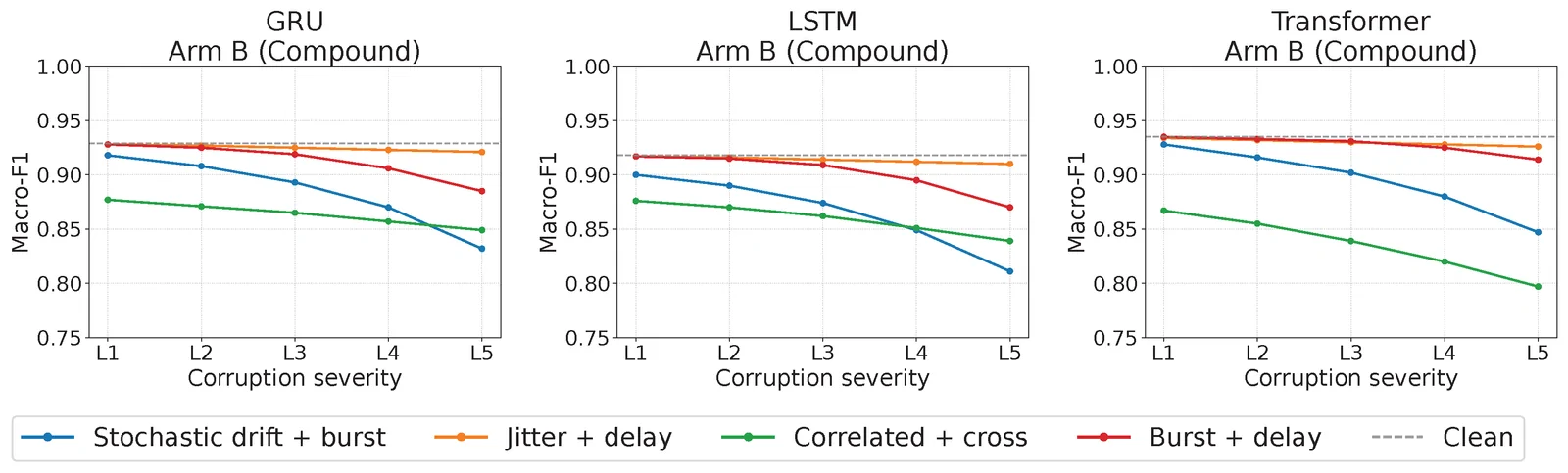
Macro-F1 versus corruption severity for noise-augmented models (Arm B) under compound (multi-fault) threats. Panels (left–right): GRU, LSTM, and Transformer Encoder. The dashed line denotes each model’s clean-test macro-F1.

This subsection evaluates models trained with evaluation matched noise augmentation i.e., Arm B. Figure 8 and Figure 9 show mean macro-F1 versus severity for atomic and compound threats, respectively, with one panel per architecture. Table 12 summarizes the macro-F1, absolute drop and relative degradation at L=5.

In contrast to the steep Arm A collapses under continuous noise, the Arm B atomic curves remained comparatively flat across L=1,…,5. Stochastic drift and correlated noise, which drove Arm A relative losses of roughly 53–79% at L=5, were largely mitigated. At L=5, Arm B relative degradation was only 7.1%, 8.0%, and 6.9% under stochastic drift for GRU, LSTM, and Transformer, respectively, and 7.2%, 5.9%, and 7.6% under correlated noise. The corresponding absolute macro-F1 values remained in the 0.84–0.87 range. *Burst & toggle* and *cross-inconsistency* curves remained close to the clean reference, with $\Delta_{\mathrm{rel}}≲2\%$. *Temporal jitter* and *temporal delay* remained essentially unaffected ($\Delta_{\mathrm{rel}}≲1\%$), similar in magnitude to the already-small Arm A timing degradations. Because Arm B was never exposed to L∈{4,5} during training, the mild high-severity slopes indicate generalization along the severity axis rather than memorization of the hardest training corruptions. Across the three panels, GRU, LSTM, and Transformer exhibited the same qualitative flattening, indicating that the benefit of evaluation matched augmentation was consistent across architectures for the atomic threats.

Compound conditions, which were never used during Arm B training, remained the most challenging setting, but degradation was substantially smaller than under Arm A. At L=5, the largest Arm B relative losses occurred for the two compounds that contain the most damaging atomic families: *stochastic drift+burst* ($\Delta_{\mathrm{rel}}=10.5\%$, 11.7%, and 9.5% for GRU, LSTM, and Transformer, respectively) and *correlated+cross* ($\Delta_{\mathrm{rel}}=8.6\%$, 8.6%, and 14.7%). The Transformer’s loss under *correlated+cross* was its largest and the largest of any model-condition pair (macro-F1 = 0.797). These values contrast with Arm A losses of roughly 63–82% on the same conditions. Absolute macro-F1 remained high, at approximately 0.81–0.85 for stochastic drift+burst and 0.80–0.85 for *correlated+cross*. *Burst+delay* produced only a modest decline ($\Delta_{\mathrm{rel}}\approx 2$–5%), while *jitter+delay* remained close to the clean baseline under Arm B ($\Delta_{\mathrm{rel}}≲1\%$), compared with about 1.7–2.1% under Arm A. These compound curves show that robustness to simultaneous multi-fault conditions improved even though Arm B was not trained on multi-fault inputs, indicating transfer from single-family augmentation to joint faults.

#### 3.2.3. Clean-Test Performance of Noise-Augmented Training (Arm B)

A common concern with noise-augmented training is that exposing the optimizer to synthetic corruptions may hurt accuracy when deployment data are clean. We therefore evaluated Arm B on the same uncorrupted held-out test split used for Arm A. This clean evaluation is distinct from the approximately 15% clean batches seen during Arm B training: it measures generalization on nominal test telemetry, not training exposure. As in the stress analyses, we reported mean macro-F1 over seven seeds and compared Arm B to Arm A with paired Wilcoxon signed-rank tests. Arm B preserved strong clean performance across architectures. GRU Arm B attained 0.929±0.005 macro-F1 versus 0.927±0.006 for Arm A (Δ=+0.003, p=0.578). LSTM was essentially unchanged (0.918±0.006 vs. 0.918±0.007, Δ=+0.000, p=0.812). The Transformer showed a small numerical decrease from 0.940±0.004 to 0.935±0.004 (Δ=−0.005, p=0.078), which did not reach significance at the conventional 0.05 level. These results indicate that evaluation matched augmentation does not trade away in-distribution accuracy for robustness. The Transformer showed a small decrease (0.005) that was not statistically significant with seven seeds. Retaining a high clean baseline is also methodologically important: absolute and relative degradation under corruption are computed from each arm’s own clean score, so Arm B’s robustness claims are not inflated by a depressed clean denominator. In operational terms, a noise-augmented model can be deployed without expecting a material penalty when telemetry is uncorrupted, while—as shown in the Arm B stress section—substantially reducing failure under continuous and multi-fault threats.

Relative degradation provides a unified contrast between the two training strategies. Whereas Arm A lost approximately two-thirds to four-fifths of its clean performance under *correlated* and *correlated+cross* threats at L=5, Arm B lost on the order of 6–15%. The largest gains occurred for the threats that dominate the Arm A failure modes, namely *correlated noise*, *stochastic drift*, and their compound combinations, whereas timing-related threats that are already mild under Arm A remained mild. This pattern is consistent with Arm B learning decision features that are more stable under continuous-feature and cross-channel perturbations rather than relying primarily on clean-manifold cues. Because clean macro-F1 was preserved while robustness gains extended to high-severity and compound conditions outside the training support, the results support evaluation matched augmentation as an effective robustness intervention.

## 4. Discussion

The results demonstrate that vehicle telematics contain sufficient temporal information to distinguish among multiple commercial automated driving systems and manual driving using deep sequence learning models. Previous studies have primarily focused on driver identification or driving-style recognition using vehicle telemetry, where temporal learning models such as LSTM have demonstrated strong performance under nominal operating conditions. In contrast, this study investigates the more challenging problem of identifying different automated driving systems across multiple manufacturers while explicitly evaluating robustness to realistic telemetry noise. Under clean evaluation, the modestly higher performance of the Transformer Encoder suggests that self-attention provides an advantage for modeling the complex temporal relationships among vehicle dynamics that characterize different ADS implementations. Unlike recurrent architectures that compress historical information into a hidden state, the Transformer Encoder directly models interactions among all observations within a sequence, allowing it to better capture coordinated temporal patterns associated with automated vehicle control. Importantly, however, this clean-data advantage does not by itself imply noise robustness: under clean training (Arm A), the Transformer remains highly sensitive to structured continuous corruptions, indicating that strong in-distribution accuracy and robustness to distribution shift are distinct properties.

The robustness analysis further clarifies what information is required for reliable ADS identification and which impairments most strongly threaten it. Clean-trained models do not degrade uniformly across threat families. The sharpest losses arise under *correlated cross-channel noise* and *stochastic drift*, together with compound conditions that embed these faults (*correlated+cross* and *stochastic drift+burst*). At high severity, relative degradation under *correlated noise* and *correlated+cross* reaches approximately 66–82% across GRU, LSTM, and Transformer Encoder models, while *stochastic drift* and *stochastic drift+burst* produce losses of roughly 53–65%. In contrast, *temporal jitter* and *temporal delay*—despite altering timing—induce only minor relative drops (typically below 2.2% at L=5). Automated driving behavior is expressed through coordinated relationships among continuous signals such as vehicle speed, acceleration, steering angle, yaw rate, and related dynamics. *Correlated noise* jointly perturbs related continuous channels and rewrites their dependence structure, moving test windows off the clean feature manifold on which Arm A decision boundaries were learned. Because perturbations are expressed in standardized units, even cross-channel perturbations with per-channel standard deviations below about 0.3 were sufficient to produce this shift. *Stochastic drift* adds measurement noise and a cumulative random walk along the window, biasing both absolute levels and apparent trends—especially near the final timestep used for labeling—thereby corrupting the features most aligned with the supervision signal. Compounds amplify these effects by pairing continuous structured noise with event *cross-inconsistency* or *burst & toggle*, removing continuous and discrete cues together. Multi-fault evaluation therefore does not create a new failure taxonomy; it amplifies the continuous-geometry sensitivity already visible in the atomic results. It also shows that strong clean macro-F1 can coexist with large losses under continuous and multi-fault conditions, and that higher in-distribution accuracy does not imply greater robustness to structured continuous perturbations.

By comparison, *temporal jitter* and *temporal delay* primarily retime otherwise intact signals through delay-with-hold or edge shifting. Amplitudes and much of the within-channel structure remain usable inside the 32-step window, so clean-trained classifiers retain most of their discriminative power. Event-level *burst & toggle* and *cross-inconsistency* produce intermediate degradation, larger than timing faults, but far smaller than *correlated* or *drift* corruption. This is consistent with the permutation results, where *gas*, *steeringAngleDeg*, and *yawRate* dominate and most binary indicators contribute less. *BrakePressed* is a notable exception, ranking first for the LSTM. Since severity is not calibrated across threat families, this link is suggestive rather than causal. Continuous dynamics are also themselves fragile when their geometry is falsified. Evaluation matched noise-augmented training (Arm B) supports this mechanistic reading: after exposure to atomic corruptions at moderate severities, relative degradation at the highest severity typically falls to about 6–15% even for previously catastrophic threats, including held-out severities and multi-fault compounds never seen during training. These results suggest that discriminative ADS information remains available under noise; the Arm A collapse is consistent with reliance on clean feature geometry. These findings emphasize that practical telemetry-based ADS identification should prioritize resilience to structured continuous and cross-channel impairments—through noise-aware training and careful sensing/communication design—rather than focusing exclusively on timing synchronization.

Although the proposed framework achieves strong classification performance using only vehicle telematics that are commonly available from the vehicle CAN bus, certain applications may require even higher reliability. Fleet management, forensic investigations, and safety-critical monitoring systems may benefit from integrating additional sensing modalities and behavioral cues. Lane-keeping behavior is one such cue: automated systems tend to exhibit stricter lane discipline than human drivers [24], and camera-based lane detection [34] could expose these differences directly. Inward-facing cameras could provide evidence of driver engagement, since models that predict where a driver looks [35] may help indicate whether a human is actively performing the driving task. In addition, the motion-planning algorithm underlying an ADS shapes its trajectories; curvature-constrained planners, for example, produce characteristic path geometries [36,37], suggesting that planner-specific signatures may help distinguish between ADS platforms. Likewise, high-precision positioning systems and HD maps could improve contextual understanding of vehicle behavior, while access to internal ADAS controller states or diagnostic information, when available, could provide software-level evidence of automated driving operation. Such multimodal fusion has the potential to improve classification robustness and reduce ambiguity in scenarios where vehicle telemetry alone exhibits overlapping characteristics between manual and automated driving, including under residual multi-fault stress that remains visible even after noise-augmented training. However, these improvements would increase hardware requirements, deployment complexity, and system cost.

A supplementary feature analysis further demonstrates that incorporating additional manufacturer-specific telemetry signals improves classification performance when such information is available. However, because these additional features are not common across all datasets, the primary evaluation is intentionally performed using the 11 shared telemetry features to ensure a fair and consistent comparison among the three ADS platforms. This observation highlights the trade-off between maximizing classification performance using richer vehicle-specific telemetry and maintaining a generalized framework that can be applied across different vehicle manufacturers.

This study has some limitations. First, the robustness evaluation is based on synthetically generated telemetry noise designed to emulate representative sensing and communication impairments, including stochastic drift, correlated cross-channel perturbations, timing faults, and event-level inconsistencies. Although these noise models capture common degradation mechanisms and enable controlled severity sweeps, they cannot fully represent the complexity of failures encountered in operational vehicles, such as intermittent hardware faults, nonlinear sensor degradation, calibration errors, packet loss, or uncontrolled combinations of impairments beyond the evaluated compound scenarios. Consequently, the reported robustness should be interpreted as performance under representative noise scenarios rather than a comprehensive assessment of all real-world operating conditions. Second, the proposed framework is evaluated using three Level 2 automated driving systems and a common subset of telemetry features available across the selected datasets. Although these datasets represent different manufacturers and software platforms, additional evaluation on a broader range of ADS platforms, vehicle models, and driving environments would further establish the generalizability of the proposed framework.

## 5. Conclusions

This paper presented a comprehensive evaluation of deep sequence learning architectures for automated driving system (ADS) identification using vehicle telematics data. Unlike approaches that rely on proprietary vehicle information or internal software states, the proposed framework identifies the active driving system solely from observable vehicle behavior. Three representative sequence learning architectures—GRU, LSTM, and Transformer Encoder—were evaluated using a large-scale heterogeneous dataset comprising Comma Openpilot, Tesla Autopilot, Cadillac Super Cruise, and manual driving scenarios. The experimental evaluation demonstrates that vehicle telematics contain sufficient temporal information to reliably distinguish among different driving systems. All three architectures achieved macro-F1s above 0.91 under clean telemetry conditions, with the Transformer Encoder providing the strongest overall clean-data classification performance.

Beyond clean accuracy, a two-arm robustness protocol was used to quantify degradation under atomic and compound telemetry threats and to assess evaluation matched noise-augmented training. Under clean training, performance did not fail uniformly: structured continuous corruptions—especially *correlated cross-channel noise* and *stochastic drift*, together with related multi-fault compounds—caused the largest absolute and relative drops in macro-F1, whereas *temporal jitter* and *temporal delay* had only minor effects. This indicates that reliable ADS identification depends critically on the integrity of continuous feature values and cross-channel dependence, not only on frame-level timing alignment. Noise-augmented training preserved clean-test accuracy while substantially reducing degradation under both in-training and held-out high-severity and compound conditions, showing that robustness can be improved without access to proprietary controller state.

In conclusion, telemetry-based ADS identification is a practical and effective approach that does not require access to proprietary vehicle software or internal controller states. The proposed framework provides a foundation for independent monitoring of automated driving systems and has potential applications in transportation safety, fleet management, cybersecurity monitoring, and regulatory verification, provided that sensing and learning pipelines account for structured continuous and cross-channel impairments that most strongly threaten sequence-based classifiers.

Future work will investigate the integration of additional sensing modalities, including camera and LiDAR observations, to further improve classification robustness under residual multi-fault stress; extend evaluation to additional ADS platforms, vehicle models, and driving environments; and complement synthetic threat models with measurements from operational telemetry faults whenever such data are available.

## 6. Previous Publications

This article extends our MILCOM 2025 conference paper [25], which introduced a GRU-based classifier that identifies Comma Openpilot, Tesla Autopilot, Cadillac Super Cruise, and manual driving from shared vehicle telematics under clean conditions. The conference paper also included a brief comparison with an LSTM and a supplementary experiment on additional vehicle features. The present article reuses the same three datasets and the 11 shared features, but differs from the conference version in the GRU architecture, the sequence length, and, most importantly, the data-splitting protocol (a chronological, segment-complete 70/15/15 split that prevents temporal leakage). All results reported here were regenerated under this new protocol.

The journal version makes the following substantive extensions:

1. Model comparison. We replace the single-GRU study with a controlled comparison of GRU, LSTM, and Transformer Encoder models under a common data pipeline, windowing protocol, and training budget, supported by 20-seed statistical testing, parameter-budget ablations, and Random Forest and XGBoost baselines.
2. Robustness framework. We introduce a structured telematics noise framework with six atomic corruption families at five severity levels and four compound multi-fault conditions, and evaluate it with a two-arm protocol: Arm A (clean training) versus Arm B (evaluation-matched noise-augmented training).
3. Failure analysis. We report absolute macro-F1 together with absolute and relative degradation against each arm’s own clean baseline, and show that structured continuous corruptions (*correlated cross-channel noise* and *stochastic drift*) dominate the failures of clean-trained models.
4. Mitigation. We show that evaluation-matched augmentation preserves clean accuracy while reducing degradation at held-out severities (L∈{4,5}) and on compound faults withheld from training.

Collectively, these additions shift the contribution from clean ADS identification alone to a joint characterization of clean performance, threat-dependent vulnerability, and noise-aware mitigation.

## Figures and Tables

**Table 1:** Comparison of Data Features Across Different Driving Systems.

Comma	Cadillac	Tesla
vEgo (m/s)	VehicleSpeed (mph)	Veh_speed (kph)
aEgo	ActVehAccel (m/s^2^)	RCM_longitudinalAccel (m/s^2^)
Gas	gasPedalandACC	Pedal_accel
yawRate	Inertial1YawRate (deg/s)	RCM_yawRate (rad/s)
standstill (0|1)	VehMovState (1|2|3|4)	veh_state_drive (0|1|2|3)
brakePressed (0|1)	BrkPedTrvlAchvd (0|1)	veh_brake_state (0|1)
brakeLights (0|1)	BrkLtsAtv (0|1)	ESP_brakeApply (0|1)
steeringAngleDeg (deg)	SteeringWheelAngle (deg)	veh_steering_angle (deg)
steeringRateDeg (deg/s)	SteeringWheelRate (deg/s)	veh_steering_speedps (D/S)
leftBlinker (0|1)	TurnSignals (0|1|2)	VCLEFT_turnSignalStatus (0|1)
rightBlinker (0|1)	TurnSignals (0|1|2)	VCRIGHT_turnSignalStatus (0|1)
steeringPressed (0|1)	LKATorqueDeliveredStatus (0|1)	No similar feature
steeringTorque	LKADriverAppldTrq (Nm)	No similar feature
brake	BrkPdlPos (% full)	No similar feature

**Table 2:** Severity-dependent parameters used in the telemetry corruption framework.

Parameter	Formula	Parameter Values	Used by
$\sigma_{\mathrm{noise}}$	0.01+0.07s	0.024, 0.038, 0.052, 0.066, 0.080	*Stochastic noise*
$\sigma_{\mathrm{drift}}$ (per-step)	0.003+0.03s	0.009, 0.015, 0.021, 0.027, 0.033	*Stochastic drift*
diag_var	0.04+0.04s	0.048, 0.056, 0.064, 0.072, 0.080	*Correlated noise*
offdiag_cov	min(0.01+0.02s,0.95diag_var)	0.014, 0.018, 0.022, 0.026, 0.030	*Correlated noise*
$p_{\mathrm{start}}$	0.005+0.08s	0.021, 0.037, 0.053, 0.069, 0.085	*Burst onset*
$p_{\mathrm{end}}$	0.25+0.35(1−s)	0.530, 0.460, 0.390, 0.320, 0.250	*Burst recovery*
$p_{\mathrm{tog}}$	0.002+0.02s	0.006, 0.010, 0.014, 0.018, 0.022	*Spurious toggle*
$p_{\mathrm{inc}}$	0.01+0.12s	0.034, 0.058, 0.082, 0.106, 0.130	*Cross-feature inconsistency*
$J,\;D_{\max}$	min(10,max(1,round(L)))	1, 2, 3, 4, 5	*Jitter shift*/*temporal delay (frames)*

**Table 3:** Telemetry corruption mechanisms, effects, and representative real-life manifestations.

Noise Type	Mechanism	Effect	Real-Life Manifestation
*Signal stochastic noise*	Additive Gaussian perturbation at each timestep, superimposed with cumulative random-walk drift	Short-term random jitter is compounded by a slowly growing offset that outgrows the noise itself over the window	Speed reading (*vEgo*) fluctuates due to vibration and then drift as sensors heat or calibration shifts over long drives
*Signal temporal jitter*	Delay-only shift of each continuous channel by Δ∼Uniform{0,…,J} with hold-at-edge fill at the window start	Observed dynamics lag true values by up to *J* frames; early-window samples are held at the first value, breaking timing alignment among continuous signals	Acceleration and steering messages arrive one to two frames late due to ECU clock skew or variable bus scheduling latency
*Signal correlated noise*	Joint multivariate perturbation of physically coupled continuous channels	Related signals are distorted together rather than independently	A CAN gateway fault or shared IMU disturbance simultaneously affects *yawRate*, *steeringAngleDeg*, and *steeringRateDeg*; a powertrain-side fault jointly perturbs *vEgo*, *aEgo*, and *gas*
*Event burst & toggle*	Markov “bad-state” process freezing a binary channel at its last value for a run of frames, plus an independent per-frame spurious bit flip	Binary event states intermittently freeze or flip unexpectedly, decoupled from the true underlying state	Turn-indicator state freezes during a stuck stalk switch or corroded connector, while brake-light bits flip intermittently from CAN frame corruption
*Event temporal delay*	Edge-triggered delay on each 0↔1 transition: holds the pre-transition value and commits the new value after a delay $\Delta ∼\mathrm{Uniform}\{0,\ldots ,D_{\max}\}$	Binary transitions appear delayed relative to the true event, while steady (non-transitioning) periods are untouched	Brake-light activation appears delayed due to gateway buffering or bus arbitration lag at the exact moment the pedal is pressed
*Event cross-feature inconsistency*	Forces one signal in a logically paired set to disagree with its partner, with fixed probability	Creates implausible combinations among signals that should be logically dependent	*brakePressed = 1* with *brakeLights = 0* (no hazard context) due to message injection or ECU desynchronization; *leftBlinker = 1* and *rightBlinker = 1* simultaneously outside a hazard event
*Stochastic drift+burst*	*Stochastic driftand burst & toggle* applied sequentially to disjoint continuous/binary channels	Sensor noise and discrete-flag corruption occur together, not in isolation	Analog sensor noise and digital CAN flag corruption can co-occur in the same window when continuous and discrete paths are stressed together
*Jitter+delay*	*Temporal jitter* and *event-delay* applied sequentially to disjoint continuous/binary channels	Continuous and discrete signals both lag their true values	Bus-timing congestion delays continuous sensor messages and discrete flag transitions together, since both share the same physical bus
*Correlated+cross*	*Correlated noise* and *cross-inconsistency* applied sequentially to disjoint channels	Two independent, coincident subsystem faults, not a shared-cause pair	A biased dynamics estimate and an unrelated cross-ECU flag desync happen to occur in the same window, without a common root cause
*Burst+delay*	*Burst & toggle* and edge *delay* applied sequentially to the same binary channels (order-sensitive)	A single discrete-signal source produces both stuck values and delayed transition reporting	A failing switch or desynchronized ECU both freezes and lags its own state, rather than exhibiting one fault type in isolation

**Table 4:** Clean-test classification performance across 20 random seeds.

Model	Accuracy	Precision (Macro)	Recall (Macro)	Macro-F1
Transformer (d=64, n_layers=6)	0.934±0.007	0.938±0.006	0.942±0.006	0.938±0.006
GRU (H=64, n_layers=6)	0.928±0.005	0.932±0.005	0.937±0.005	0.932±0.005
LSTM (H=64, n_layers=6)	0.913±0.007	0.919±0.006	0.923±0.007	0.918±0.007

**Table 5:** Class-wise precision, recall, and F1 (mean over 20 seeds). Bold values within each model indicate the highest precision, recall, and F1-score across classes; bold F1-scores across models indicate the highest F1-score for each class.

Class	GRU			LSTM			Transformer		
	P	R	F1	P	R	F1	P	R	F1
Openpilot	0.878	0.979	0.925	0.859	0.972	0.912	0.890	0.981	**0.933**
Manual	0.946	0.811	0.873	0.941	0.772	0.848	0.950	0.823	**0.882**
Autopilot	0.923	0.968	0.945	0.901	0.965	0.932	0.924	0.970	**0.947**
**Super Cruise**	**0.982**	**0.989**	**0.985**	**0.975**	**0.983**	**0.979**	**0.987**	**0.993**	**0.990**
Macro Avg	0.932	0.937	0.932	0.919	0.923	0.918	0.938	0.942	0.938

**Table 6:** Clean-test performance comparison between conventional machine-learning and sequence models over 20 random seeds. Bold values indicate the highest mean performance for each evaluation metric across all models.

Model	Accuracy	Precision (Macro)	Recall (Macro)	Macro-F1
Random Forest	0.9035±0.0002	0.9090±0.0001	0.9139±0.0001	0.9078±0.0002
XGBoost	0.9136±0.0003	0.9196±0.0003	0.9246±0.0003	0.9179±0.0003
LSTM (H=64)	0.913±0.007	0.919±0.006	0.923±0.007	0.918±0.007
GRU (H=64)	0.928±0.005	0.932±0.005	0.937±0.005	0.932±0.005
Transformer (d=64)	0.934±0.007	0.938±0.006	0.942±0.006	0.938±0.006

**Table 7:** Comparison of model complexity, computational cost, and clean-test performance (iso-depth/iso-width baselines, mean over 20 seeds). Train GPU is peak memory during training; Infer GPU is peak memory during batch-1 inference. Lat. is batch-1 wall-clock time.

Model	Params	FLOPs	Size	Train (s)	Lat. (ms)	Train GPU	Infer GPU	Macro-F1
GRU	140 k	8.8 M	0.56 MB	932±96	0.74	163 MB	25 MB	0.932
LSTM	186 k	11.7 M	0.75 MB	812±90	0.90	158 MB	28 MB	0.918
Transformer	301 k	20.5 M	1.24 MB	1098±105	1.44	270 MB	34 MB	0.938

**Table 8:** Parameter-budget ablation results (mean over 20 seeds; same split and training protocol). Train (s) is early-stopped wall-clock time; Epochs is the mean number of completed epochs.

Model	Params	FLOPs	Train (s)	Epochs	GPU (MB)	Latency (ms)	Macro-F1
GRU, H=64	139,844	8.79 M	932	22.4	163	0.74	0.932±0.005
GRU, H=94	∼298 k	18.86 M	697	16.5	247	0.68	0.930±0.007
LSTM, H=64	186,372	11.72 M	812	18.9	158	0.90	0.918±0.007
LSTM, H=82	∼304 k	19.17 M	733	16.9	217	0.68	0.915±0.009
Transformer, d=64	300,932	20.49 M	1098	11.6	270	1.44	0.938±0.006
Transformer, d=48	∼170 k	11.83 M	1397	13.2	281	1.35	0.936±0.007

**Table 9:** Top-4 features ranked by permutation importance (macro-F1 drop) averaged over the three architectures, on the clean test set. Per-model values are shown; larger values indicate stronger reliance. A feature’s rank within an individual model may differ.

Rank	Feature	GRU	LSTM	Transformer
1	gas	0.6606	0.6031	0.6125
2	steeringAngleDeg	0.3809	0.5143	0.4795
3	brakePressed	0.3792	0.6377	0.3444
4	yawRate	0.3267	0.5317	0.4279

**Table 10:** Classification result of GRU with 14 features.

Type of Driving	Precision	Recall	F1-Score
Comma Openpilot	0.94	0.98	0.97
Manual Driving	0.97	0.92	0.94
Cadillac Super Cruise	0.99	0.99	0.99
Macro Avg	0.97	0.97	0.97

**Table 11:** Arm A robustness at L=5 (7-seed means): macro-F1/$\Delta_{\mathrm{abs}}$/$\Delta_{\mathrm{rel}}$ (%).

Threat	GRU	LSTM	Transformer
Stochastic drift	0.427/0.500/54.0	0.429/0.489/53.3	0.390/0.550/58.5
Temporal jitter	0.912/0.014/1.6	0.903/0.015/1.6	0.922/0.018/2.0
Correlated noise	0.305/0.621/67.0	0.310/0.608/66.2	0.196/0.744/79.1
Burst & toggle	0.666/0.261/28.1	0.685/0.233/25.4	0.770/0.170/18.1
Temporal delay	0.925/0.001/0.2	0.916/0.002/0.2	0.939/0.001/0.1
Cross-inconsistency	0.667/0.260/28.0	0.664/0.254/27.7	0.739/0.201/21.4
Stochastic drift+burst	0.329/0.597/64.4	0.342/0.575/62.7	0.325/0.615/65.4
Jitter+delay	0.911/0.015/1.7	0.901/0.016/1.8	0.921/0.019/2.1
Correlated+cross	0.276/0.650/70.2	0.297/0.621/67.6	0.170/0.770/81.9
Burst+delay	0.577/0.350/37.7	0.587/0.331/36.1	0.711/0.229/24.4

**Table 12:** Arm B robustness at L=5 (seven-seed means). Values are reported as macro-F1/$\Delta_{\mathrm{abs}}$/$\Delta_{\mathrm{rel}}$ (%).

Threat	GRU	LSTM	Transformer
Stochastic drift	0.863/0.066/7.1	0.844/0.074/8.0	0.871/0.064/6.9
Temporal jitter	0.922/0.007/0.8	0.911/0.007/0.8	0.926/0.009/1.0
Correlated noise	0.862/0.067/7.2	0.864/0.054/5.9	0.864/0.071/7.6
Burst & toggle	0.912/0.018/1.9	0.900/0.018/2.0	0.926/0.010/1.0
Temporal delay	0.928/0.001/0.1	0.917/0.001/0.1	0.935/0.001/0.1
Cross-inconsistency	0.923/0.006/0.7	0.911/0.007/0.8	0.930/0.006/0.6
Stochastic drift+burst	0.832/0.097/10.5	0.811/0.107/11.7	0.847/0.089/9.5
Jitter+delay	0.921/0.008/0.8	0.910/0.008/0.8	0.926/0.009/1.0
Correlated+cross	0.849/0.080/8.6	0.839/0.079/8.6	0.797/0.138/14.7
Burst+delay	0.885/0.045/4.8	0.870/0.048/5.2	0.914/0.021/2.2

## Data Availability

All of our datasets will be available through Platform for Innovative Vehicle Open Telematics (PIVOT) project [38].

## References

[1] Tesla Autopilot and Full Self-Driving Capability. https://www.tesla.com/support/autopilot.

[2] Cadillac Super Cruise—Hands-Free Driving. https://www.cadillac.com/technology/super-cruise.

[3] Comma.ai Openpilot. https://github.com/commaai/openpilot.

[4] Keysight Technologies What Is a Software-Defined Vehicle?. https://www.keysight.com/us/en/cmp/topics/what-is-a-software-defined-vehicle.html.

[5] Wikipedia Volkswagen Emissions Scandal. https://en.wikipedia.org/wiki/Volkswagen_emissions_scandal.

[6] Hochreiter S., Schmidhuber J. (1997). Long Short-Term Memory. Neural Comput..

[7] Cho K., van Merriënboer B., Gulcehre C., Bahdanau D., Bougares F., Schwenk H., Bengio Y. (2014). Learning Phrase Representations Using RNN Encoder-Decoder for Statistical Machine Translation. arXiv.

[8] Vaswani A., Shazeer N., Parmar N., Uszkoreit J., Jones L., Gomez A.N., Kaiser L., Polosukhin I. Attention Is All You Need. Proceedings of the Advances in Neural Information Processing Systems 30 (NeurIPS 2017).

[9] Saleh K., Hossny M., Nahavandi S. Driving Behavior Classification Based on Sensor Data Fusion Using LSTM Recurrent Neural Networks. Proceedings of the 2017 IEEE 20th International Conference on Intelligent Transportation Systems (ITSC).

[10] Enev M., Takakuwa A., Koscher K., Kohno T. (2016). Automobile Driver Fingerprinting. Proc. Priv. Enhancing Technol..

[11] Girma A., Yan X., Homaifar A. Driver Identification Based on Vehicle Telematics Data Using LSTM-Recurrent Neural Network. Proceedings of the 2019 IEEE 31st International Conference on Tools with Artificial Intelligence (ICTAI).

[12] Carvalho E., Ferreira B.V., Ferreira J., de Souza C., Carvalho H.V., Suhara Y., Pentland A.S., Pessin G. Exploiting the Use of Recurrent Neural Networks for Driver Behavior Profiling. Proceedings of the 2017 International Joint Conference on Neural Networks (IJCNN).

[13] Guo B., Hansen J.H.L. T-EnFP: An Efficient Transformer Encoder-Based System for Driving Behavior Classification. Proceedings of the 2024 IEEE International Conference on Acoustics, Speech and Signal Processing (ICASSP).

[14] Biddle L., Fallah S. (2021). A Novel Fault Detection, Identification and Prediction Approach for Autonomous Vehicle Controllers Using SVM. Automot. Innov..

[15] Abboush M., Knieke C., Rausch A. (2025). Advancing Real-Time Validation of Automotive Software Systems via Continuous Integration and Intelligent Failure Analysis. Sci. Rep..

[16] Abboush M., Bamal D., Knieke C., Rausch A. (2022). Intelligent Fault Detection and Classification Based on Hybrid Deep Learning Methods for Hardware-in-the-Loop Test of Automotive Software Systems. Sensors.

[17] Shahriar M.H., Al Barat M.M., Sundar H., Ramakrishnan N., Hou Y.T., Lou W. (2025). On the Fragility of Multimodal Perception to Temporal Misalignment in Autonomous Driving. arXiv.

[18] Fan M., Zuo Y., Blaes P., Montgomery H., Das S. (2025). Robust Sensor Fusion Against On-Vehicle Sensor Staleness. arXiv.

[19] Zhang Z., Jin Z., Peng Y., Guo X., Liu H., Zhang S., Ma X., Wu Z., Yan J., Jia X. (2026). Bench2Drive-Robust: Benchmarking Closed-Loop Autonomous Driving Under Deployment Perturbations. arXiv.

[20] Canevaro A., Yu H., Schmidt J., Li P., Lindner S., Stork W., Martius G., Jordan J. (2026). Shift & Drift: A Zero-Shot Benchmark for Generalizable and Robust Autonomous Driving Motion Planning. arXiv.

[21] Wei J., Li Y., Chen B., Zhao Y., Li H., Yao D., Lu B., Ye X., Feng J., Im S.K. (2026). Benchmarking Inductive Biases for Multivariate Time-Series Anomaly Detection with a Robust Multi-View Channel-Graph Detector. arXiv.

[22] Hu H., Ai X. (2026). Exogenous Dropout: A Simple, Strong Baseline for Corruption-Robust Time Series Forecasting with Covariates. arXiv.

[23] Maresca F., Grazioli F., Albanese A., Sciancalepore V., Negri G., Costa-Perez X. Are You a Robot? Detecting Autonomous Vehicles from Behavior Analysis. Proceedings of the 2024 IEEE International Conference on Robotics and Automation (ICRA).

[24] Li X., Wang T., Jiao J., Claudel C., Guo Z. (2025). SVBRD-LLM: Self-Verifying Behavioral Rule Discovery for Autonomous Vehicle Identification. arXiv.

[25] Shrestha B., Acharya B., Petit J., Papadopoulos C. Classification of Automated Driving Systems. Proceedings of the MILCOM 2025—2025 IEEE Military Communications Conference (MILCOM).

[26] Schafer H., Santana E., Haden A., Biasini R. (2018). A Commute in Data: The comma2k19 Dataset. arXiv.

[27] Duoba M., Baby T.V. Cadillac CT6 Super Cruise On-Road Data; Dataset; February 2025. https://www.osti.gov/biblio/1922210.

[28] Duoba M., Baby T.V. Tesla Model 3 Autopilot On-Road Data; Dataset; February 2025. https://www.osti.gov/biblio/1922211.

[29] Christ M., Braun N., Neuffer J., Kempa-Liehr A.W. tsfresh. https://tsfresh.readthedocs.io/en/latest/.

[30] Vergara J.R., Estévez P.A. (2014). A Review of Feature Selection Methods Based on Mutual Information. Neural Comput. Appl..

[31] Hendrycks D., Dietterich T. (2019). Benchmarking Neural Network Robustness to Common Corruptions and Perturbations. arXiv.

[32] Breiman L. (2001). Random Forests. Mach. Learn..

[33] Chen T., Guestrin C. XGBoost: A Scalable Tree Boosting System. Proceedings of the 22nd ACM SIGKDD International Conference on Knowledge Discovery and Data Mining.

[34] Zhang Y., Tu Z., Lian W., Hu Y., Sun S., Xiao Y., Cheng Y. (2026). BANet: Bidirectional Feature Aggregation and Adaptive Multi-Scene Perception-Based Lane Detection for Autonomous Driving. IEEE Trans. Intell. Transp. Syst..

[35] Zhang Y., Zhang Y., Kang Y., Xiao Y., Wang T. (2026). Multi-Scale Feature Reconstruction and Gated Dual-Attention Network for Driver Attention Prediction. Appl. Soft Comput..

[36] Zhou X., Li L., Zhang X., Gao H., Yao K., Xu X. (2025). A Unified and Quality-Guaranteed Approach for Dubins Vehicle Path Planning With Obstacle Avoidance and Curvature Constraint. IEEE Trans. Intell. Transp. Syst..

[37] Zhou X., Li L., Zhang X., Jin S., Xu X., Shi D., Wang H. (2026). A Seventy-Year Review of Dubins Robot Path Planning: Basics, Developments and Opportunities. Ind. Robot.

[38] PIVOT Project https://pivot-auto.org/.

